# Climate change and ecosystem shifts in the southwestern United States

**DOI:** 10.1038/s41598-023-46371-x

**Published:** 2023-11-15

**Authors:** Grant M. Harris, Steven E. Sesnie, David R. Stewart

**Affiliations:** United States Fish and Wildlife Service, Albuquerque, NM USA

**Keywords:** Climate sciences, Ecology

## Abstract

Climate change shifts ecosystems, altering their compositions and instigating transitions, making climate change the predominant driver of ecosystem instability. Land management agencies experience these climatic effects on ecosystems they administer yet lack applied information to inform mitigation. We address this gap, explaining ecosystem shifts by building relationships between the historical locations of 22 ecosystems (c. 2000) and abiotic data (1970–2000; bioclimate, terrain) within the southwestern United States using ‘ensemble’ machine learning models. These relationships identify the conditions required for establishing and maintaining southwestern ecosystems (i.e., ecosystem suitability). We projected these historical relationships to mid (2041–2060) and end-of-century (2081–2100) periods using CMIP6 generation BCC-CSM2-MR and GFDL-ESM4 climate models with SSP3-7.0 and SSP5-8.5 emission scenarios. This procedure reveals how ecosystems shift, as suitability typically increases in area (~ 50% (~ 40% SD)), elevation (12–15%) and northing (4–6%) by mid-century. We illustrate where and when ecosystems shift, by mapping suitability predictions temporally and within 52,565 properties (e.g., Federal, State, Tribal). All properties had ≥ 50% changes in suitability for ≥ 1 ecosystem within them, irrespective of size (≥ 16.7 km^2^). We integrated 9 climate models to quantify predictive uncertainty and exemplify its relevance. Agencies must manage ecosystem shifts transcending jurisdictions. Effective mitigation requires collective action heretofore rarely instituted. Our procedure supplies the climatic context to inform their decisions.

## Introduction

Climate change shifts ecosystems^1–4^. Plant assemblages defining ecosystems require specific abiotic conditions, so as climate change alters precipitation and temperature patterns beyond historical ranges, ecosystems track the bioclimatic transitions^3–6^. Globally, land management agencies, conservation organizations, and indigenous communities recognize these climate-induced shifts on ecosystems within their jurisdictional properties yet struggle to develop informed responses. Few options exist, broadly categorized as resisting, accepting, or directing (i.e., facilitating) climate induced changes^7,8^. Given the shortfalls in understanding relationships between climate change and ecosystems, and the lack of maps predicting future ecosystem distributions at relevant scales, resisting or accepting climate change become default approaches. This information gap throttles the implementation of applied, on-the-ground mitigation strategies for addressing the effects of climate change on ecosystems within most jurisdictional lands^9^. Designing and enacting deliberative approaches for managing ecosystems requires data describing ecosystem relationships with climate, how ecosystems are likely to respond as climate changes, followed by predictions describing where and when ecosystem shifts are likely to occur at the scales land-based organizations work.

**Figure 1:**
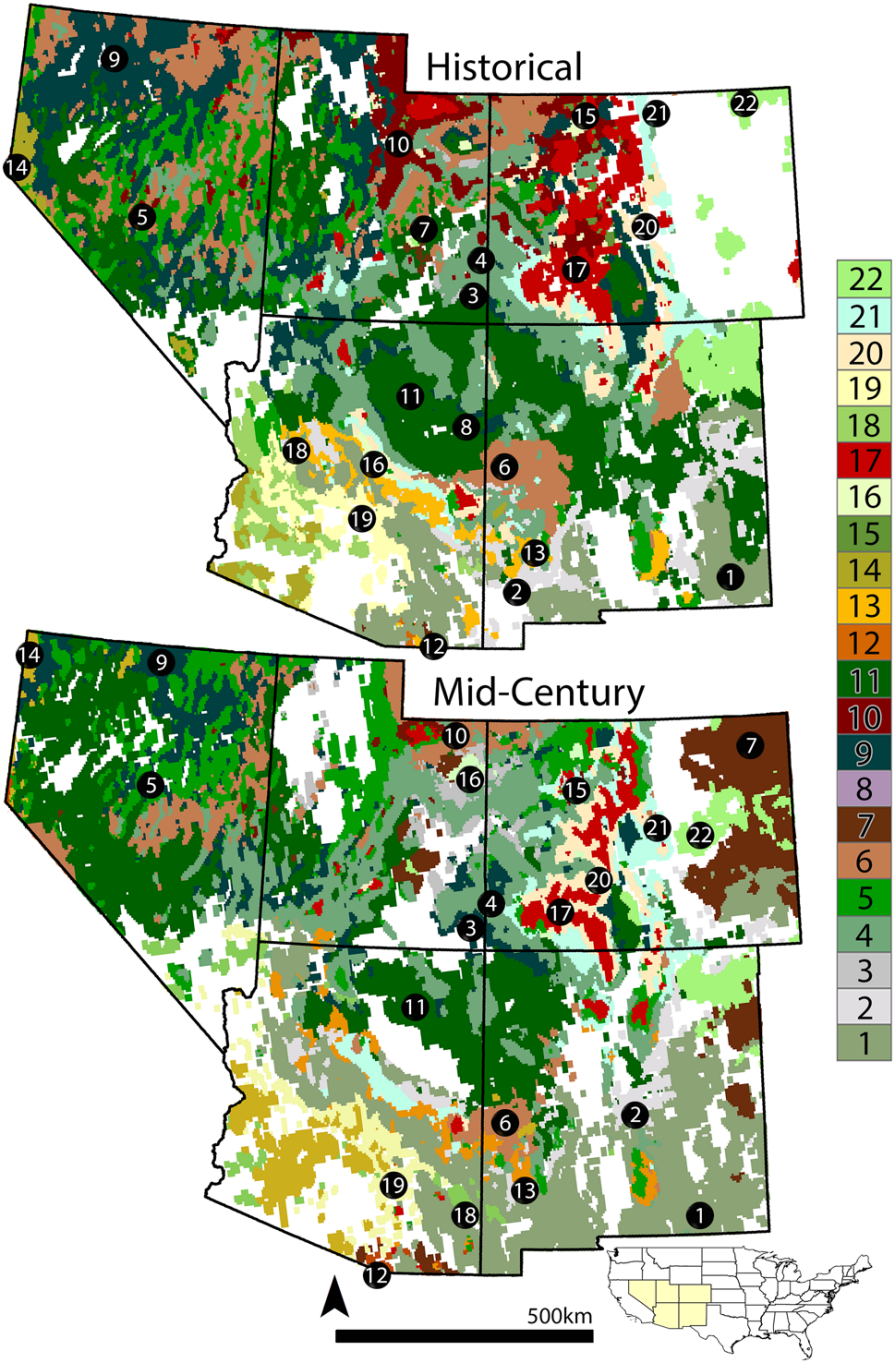
Historical (1970–2000) and mid-century (2041–2080) predictions of ecosystem suitability for 22 ecosystems within the southwestern United States (inset; states clockwise from upper left: Nevada, Utah, Colorado, New Mexico, Arizona (suitability values ≥ 0.5; coarse depictions)). These predictions originate from relationships between 19 downscaled bioclimatic variables (WorldClim v2.1 2.5-min grids), three terrain variables, and the historical geographical locations of each ecosystem using the USGS National GAP Analysis Program. These relationships identify the combinations of abiotic variables that best predict the presence of each ecosystem and therefore, the suitability of each grid cell (16.7 km^2^) to establish and maintain a given ecosystem (historical prediction). We evaluated these models using spatial projections of the bioclimate variables with climate models to predict mid-century ecosystem suitability, given each ecosystem’s relationship with the abiotic variables (BCC-CSM2-MR SSP-3.70 depicted). The numbers correspond to the following ecosystems: 1—Apacherian-Chihuahuan Mesquite Upland Scrub, 2—Apacherian-Chihuahuan Semi-Desert Grassland and Steppe, 3—Colorado Plateau Pinyon Juniper Shrublands, 4—Colorado Plateau Pinyon Juniper Woodland, 5—Great Basin Pinyon-Juniper Woodland, 6—Great Basin Xeric Mixed Sagebrush Shrubland, 7—Inter-Mountain Basins Active and Stabilized Dune, 8—Inter-Mountain Basins Juniper Savanna, 9—Inter-Mountain Basins Big Sagebrush Shrubland, 10—Inter-Mountain Basins Montaine Big Sagebrush Steppe, 11—Inter-Mountain Basins Semi-Desert Shrub-Steppe, 12—Madrean Encinal, 13—Madrean Pinyon Juniper Woodland, 14—North American Warm Desert Lower Montane Riparian Woodland and Shrubland, 15—Rocky Mountain Lodgepole Pine Forest, 16—Rocky Mountain Lower Montane-Foothill Riparian Woodland and Shrubland, 17—Rocky Mountain Subalpine Dry-Mesic Spruce-Fir Forest and Woodland, 18—Sonora-Mojave Creosote-White Bursage Desert Scrub, 19—Sonoran Paloverde-Mixed Cacti Desert Scrub, 20—Southern Rocky Mountain Dry-Mesic Montane Mixed Conifer Forest and Woodland, 21—Southern Rocky Mountain Ponderosa Pine Woodland, 22—Western Great Plains Shortgrass Prairie (R version 4.2.3 https://www.r-project.org/; ArcGIS Desktop 10.8.1 http://www.esri.com).

We demonstrate how to address this information gap by modeling, quantifying and mapping the climatic effects on ecosystems in the southwestern United States (Arizona, Colorado, Nevada, New Mexico and Utah; Fig. 1). Throughout the southwest, climate projections indicate increasing temperatures and altered precipitation regimes likely to engender pronounced ecosystem changes^10–12^. Climate change is also exacerbating disturbance events and weakening the resiliency of southwestern ecosystems, hastening ecosystem transitions^6,13–15^.

Our approach projects the suitability of a given location for establishing and maintaining ecosystem types (hereafter termed “ecosystem suitability”), by building relationships between the locations of 22 southwestern ecosystems (c. 2000) and abiotic data (1970–2000) occurring within those ecosystems, using ‘ensemble’ machine learning (ML) models (Fig. 1)^16^. Ecosystem information relied on plot surveys conducted by the USGS National Gap Analysis Program, with bioclimate forming the bulk of abiotic data^17^. Our work focused solely on the abiotic contributions to ecosystem suitability and did not address other ecophysiological factors.

We projected these relationships to predict suitability at mid-century (2041–2060) and end-of-century (2081–2100) using BCC-CSM2-MR and GFDL-ESM4 climate change models (GCM) from the Coupled Model Intercomparison Projects generation 6 (CMIP6) with two Shared Socioeconomic Pathway (SSP) emission scenarios. We used SSP3-7.0 and SSP5-8.5 for BCC-CSM2-MR and SSP3-7.0 with GFDL-ESM4. The SSP3-7.0 scenario omits any future climate policy changes, has high greenhouse gas emissions (doubling of current levels by 2100), and a warming of ~ 3.6 °C by 2100^18^. The SSP5-8.5 scenario also excludes further climate policy, includes very high greenhouse gas emissions (doubling of current levels by 2050) with global warming of ~ 4.4 °C by 2100^18^. We also included SSP2-4.5 when projecting ecosystem suitability and quantifying predictive uncertainty for the Colorado Pinyon Juniper Woodland ecosystem using a multiple GCM example. The SSP2-4.5 scenario considers CO^2^ emissions remaining at current levels until mid-century with global warming of ~ 2.7 °C by 2100^18^. SSP5-8.5 forms a high boundary of climatic possibility, followed by SSP3-7.0 and SSP2-4.5, with the feasibility or likelihood of individual scenarios in debate^19,20^. We did not include the SSP1-1.9 and SSP1-2.6 scenarios that incorporate strong mitigation for greenhouse gas emissions, as these scenarios are either unrealistic or soon to be^18^.

We use these projections to quantify “how” ecosystem suitability changes across the southwest by examining temporal changes in the amount of suitable area, plus elevation and northing deviations over time. We examine the distribution of suitability values within each ecosystem, by comparing the amount of area within low (< 0.25) and high suitability (≥ 0.5) to further diagnose an ecosystems’ potential to transition. Locations with low ecosystem suitability suggest areas approaching ‘tipping points’, whereby one ecosystem shifts out as another transitions in^21–23^.

Land-management agencies need data describing “when” and “where” ecosystem suitability changes within the jurisdictions they administer to inform mitigation strategies. Therefore, we quantified and mapped ecosystem suitability across time and geographical space (16.7 km^2^ resolution), within state, jurisdiction, and individual properties, including locations Tribal, private, State or Federally owned (e.g., National Parks, National Wildlife Refuges, National Forests [*n* = 52,565 properties]).

Quantifying temporal and spatial uncertainty in climate model predictions forms an important part of the modeling and decision-making process, by further informing where, when and how to conduct mitigation for climatic changes occurring on ecosystems. We demonstrated this procedure and exemplified the utility of results by incorporating nine GCMs and three emission scenarios to predict ecosystem suitability and accompanying uncertainty metrics for the Colorado Plateau Pinyon Juniper Woodland. We summarized results within state and jurisdictional boundaries to illustrate how knowledge of the climate context shapes mitigation strategies. For instance, geographical locations having low ecosystem suitability and uncertainty reveal places where that ecosystem is unlikely to resist severe disturbances and return to its original state^14^, making these places strong candidates for accepting ecosystem shifts. Other areas gaining ecosystem suitability with low uncertainty are more apt to resist climate change, especially if the suitability increases trend positively through time. In application, we prefer that the selection of GCMs and emission scenarios be partner based, thereby bridging the knowledge of managers and modelers, so underlying model assumptions align with project goals and the products produced inform those steering mitigation design.

The methods we exemplify provide data to empower agencies in building regional, collaborative mitigation strategies that transcend their jurisdictional boundaries. Given the scope and scale of climatic changes on ecosystem suitability throughout the southwest, such regional, coordinated mitigation actions offer the best chances of success.

## Results

We established relationships between 22 abiotic variables (historical period, 1970–2000) and the locations of 22 ecosystems within the southwestern USA, to identify the combination of abiotic variables, and their relative importance, in predicting the suitability of a given location (pixel) for the establishment and persistence of each ecosystem. We evaluated models, considering those with area under the curve (AUC) < 0.75 and Sørensen similarity index < 0.5 having lower performance (Table 1)^24,25^.

**Table 1:** Ecosystems within the southwestern United States (Arizona, Colorado, Nevada, New Mexico, Utah;* n* = 22) classified at the U.S. National Vegetation Classification (NVC) group level.

Ecological system	Total	Training	Testing	AUC	SOR
Apacherian-Chihuahuan Mesquite Upland Scrub	665	571	94	0.83	0.55
Apacherian-Chihuahuan Semi-Desert Grassland and Steppe	443	389	54	0.82	0.47
Colorado Plateau Pinyon Juniper Shrublands	312	247	65	0.82	0.5
Colorado Plateau Pinyon Juniper Woodland	2345	2124	221	0.88	0.67
Great Basin Pinyon-Juniper Woodland	1200	986	214	0.85	0.71
Great Basin Xeric Mixed Sagebrush Shrubland	1454	1166	288	0.79	0.76
Inter-Mountain Basins Active and Stabilized Dune	154	115	39	0.82	0.14
Inter-Mountain Basins Big Sagebrush Shrubland	3362	2704	658	0.82	0.85
Inter-Mountain Basins Juniper Savanna	138	110	28	0.8	0.13
Inter-Mountain Basins Montaine Big Sagebrush Steppe	677	530	147	0.9	0.72
Inter-Mountain Basins Semi-Desert Shrub-Steppe	2158	1740	418	0.77	0.73
Madrean Encinal	58	50	8	0.78	0.22
Madrean Pinyon Juniper Woodland	364	301	63	0.89	0.65
North American Warm Desert Lower Montane Riparian Woodland and Shrubland	150	123	27	0.76	0.27
Rocky Mountain Lower Montane-Foothill Riparian Woodland and Shrubland	146	119	27	0.79	0.11
Rocky Mountain Subalpine Dry-Mesic Spruce-Fir Forest and Woodland	329	251	78	0.95	0.65
Rocky Mountain Lodgepole Pine Forest	157	129	28	0.91	0.43
Sonora-Mojave Creosote-White Bursage Desert Scrub	419	331	88	0.89	0.62
Sonoran Paloverde-Mixed Cacti Desert Scrub	483	390	93	0.91	0.61
Southern Rocky Mountain Dry-Mesic Montane Mixed Conifer Forest and Woodland	492	390	102	0.95	0.72
Southern Rocky Mountain Ponderosa Pine Woodland	1113	882	231	0.96	0.83
Western Great Plains Shortgrass Prairie	389	308	81	0.92	0.61

We used ensemble modeling to predict ecosystem suitability at the historic period (1970–2000) based on ecosystem location with bioclimate and terrain variables. We projected these models to generate ecosystem suitability predictions at mid-century (1941–1960) and end-of-century (2081–2100) periods using CMIP6 BCC-CSM2-MR and GFDL-ESM4 climate models with SSP3-7.0 and SSP5-8.5 emission scenarios. Columns report the number of field plots analyzed to build the historical models (Total), number of plots used for model training (Training), testing (Testing), plus area under the curve (AUC) and Sørensen similarity index (SOR) metrics used for measuring model performance.

Across all models, maximum temperature during the warmest month (bio5) had the greatest influence on predicting an ecosystem’s historical distribution, being the most selected bioclimatic variable with the highest importance values (based on Root Mean Square Error (RMSE), Table 2)). Elevation occurred in all models (likely indicating unexplained variation associated with site biophysical factors), while transformed aspect had the highest variable importance, and therefore greatest predictive influence (Table 2). The amount of precipitation occurring in the driest month (bio14) had the lowest mean variable importance (i.e., least influence) and occurred in fewest models (*n* = 13; Table 2).

**Table 2:** All predictor variables used in ensemble models to predict ecosystem suitability, including 19 Worldclim bioclimate variables (https://www.worldclim.org/data/bioclim.html), and 3 terrain variables built from the 90 m Shuttle Radar Topography Mission Digital Elevation Model (SRTM DEM; “trasp” = transformed aspect).

Variable	Description	Freq.	RMSE	SD
bio1	Annual Mean Temperature	20	0.229	0.07
bio2	Mean Diurnal Range (Mean of monthly (max temp—min temp))	19	0.225	0.059
bio3	Isothermality (BIO2/BIO7) (× 100)	14	0.224	0.054
bio4	Temperature Seasonality (standard deviation × 100)	21	0.227	0.065
bio5	Max Temperature of Warmest Month	21	0.247	0.065
bio6	Min Temperature of Coldest Month	18	0.231	0.06
bio7	Temperature Annual Range (BIO5-BIO6)	21	0.226	0.064
bio8	Mean Temperature of Wettest Quarter	19	0.232	0.067
bio9	Mean Temperature of Driest Quarter	20	0.234	0.073
bio10	Mean Temperature of Warmest Quarter	18	0.242	0.073
bio11	Mean Temperature of Coldest Quarter	17	0.235	0.069
bio12	Annual Precipitation	21	0.238	0.071
bio13	Precipitation of Wettest Month	18	0.230	0.071
bio14	Precipitation of Driest Month	13	0.213	0.064
bio15	Precipitation Seasonality (Coefficient of Variation)	21	0.234	0.072
bio16	Precipitation of Wettest Quarter	20	0.238	0.073
bio17	Precipitation of Driest Quarter	20	0.232	0.072
bio18	Precipitation of Warmest Quarter	21	0.231	0.068
bio19	Precipitation of Coldest Quarter	19	0.233	0.064
elevation	SRTM 90 m elevation data	22	0.242	0.072
slope	Degrees slope from SRTM DEM	18	0.242	0.065
trasp	Transformed aspect = 1−cos((π/180) * (aspect-30))/2	18	0.251	0.071

The mean and standard deviation (SD) values identify variable importance across all 22 ecosystems within the southwestern United States (Arizona, Colorado, Nevada, New Mexico, Utah). Variable importance was measured as the increase in Root Mean Square Error (RMSE) with the variable removed from the machine learning ensemble model, to estimate the amount that each predictor variable contributed to the ecosystem suitability predictions (n = 35 permutations). The column “Freq.” indicates the number of times (frequency) the variable was selected with recursive feature elimination (RFE) for predicting an ecosystems suitability.

**Figure 2:**
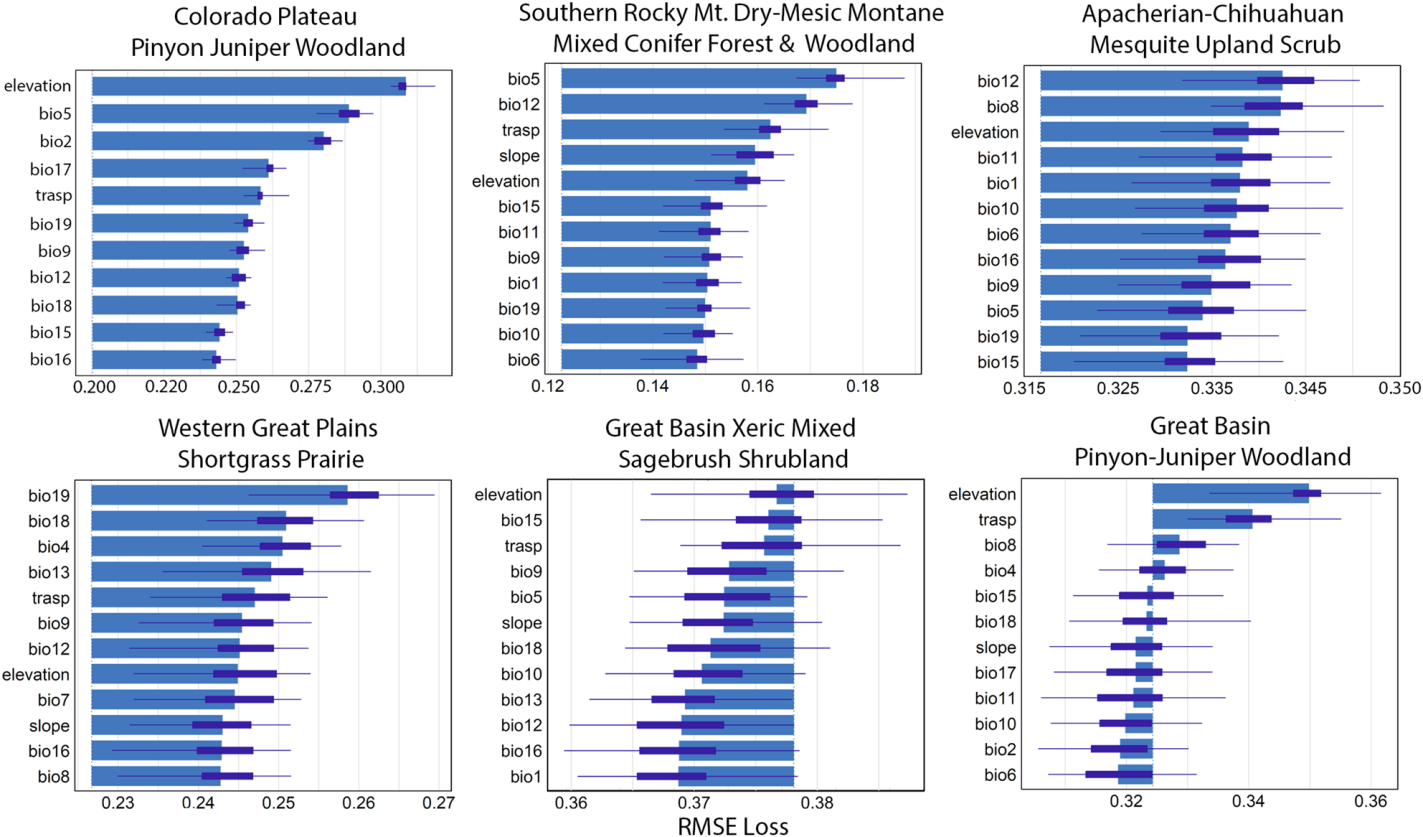
Top set of 12 bioclimatic and terrain variables best predicting the suitability of a location (16.7 km^2^ pixel) to establish and maintain six example ecosystems in the southwestern United States (Arizona, Colorado, Nevada, New Mexico, Utah). The variable “trasp” indicates transformed aspect. These results are generated from an ensemble model using the historical climate data (1970–2000). The horizontal axis displays the increase in root mean square error (RMSE) after removing each variable from the ensemble model for 35 random data permutations. The purple box plots indicate the range and quartile values of the RMSE for each model permutation. The mean values are indicated by the end of the blue horizontal bars intersecting with the purple box plots. Colorado Plateau Pinyon Juniper Woodland had 11 total predictor variables based on the results from the random forest optimization run performed prior to the ensemble modelling step.

Predictions of ecosystem suitability rely on complex interactions among all the abiotic variables, although the most influential variables that associated with each ecosystem’s historical presence varied by ecosystem type (Fig. 2). Some ecosystems, like the Great Basin Pinyon-Juniper Woodland, displayed clear patterns in variable importance, while others such as Apacherian-Chihuahuan Mesquite Upland Scrub relied more on a diverse combination of terrain and bioclimatic variables with comparable importance levels (Fig. 2).

**Figure 3:**
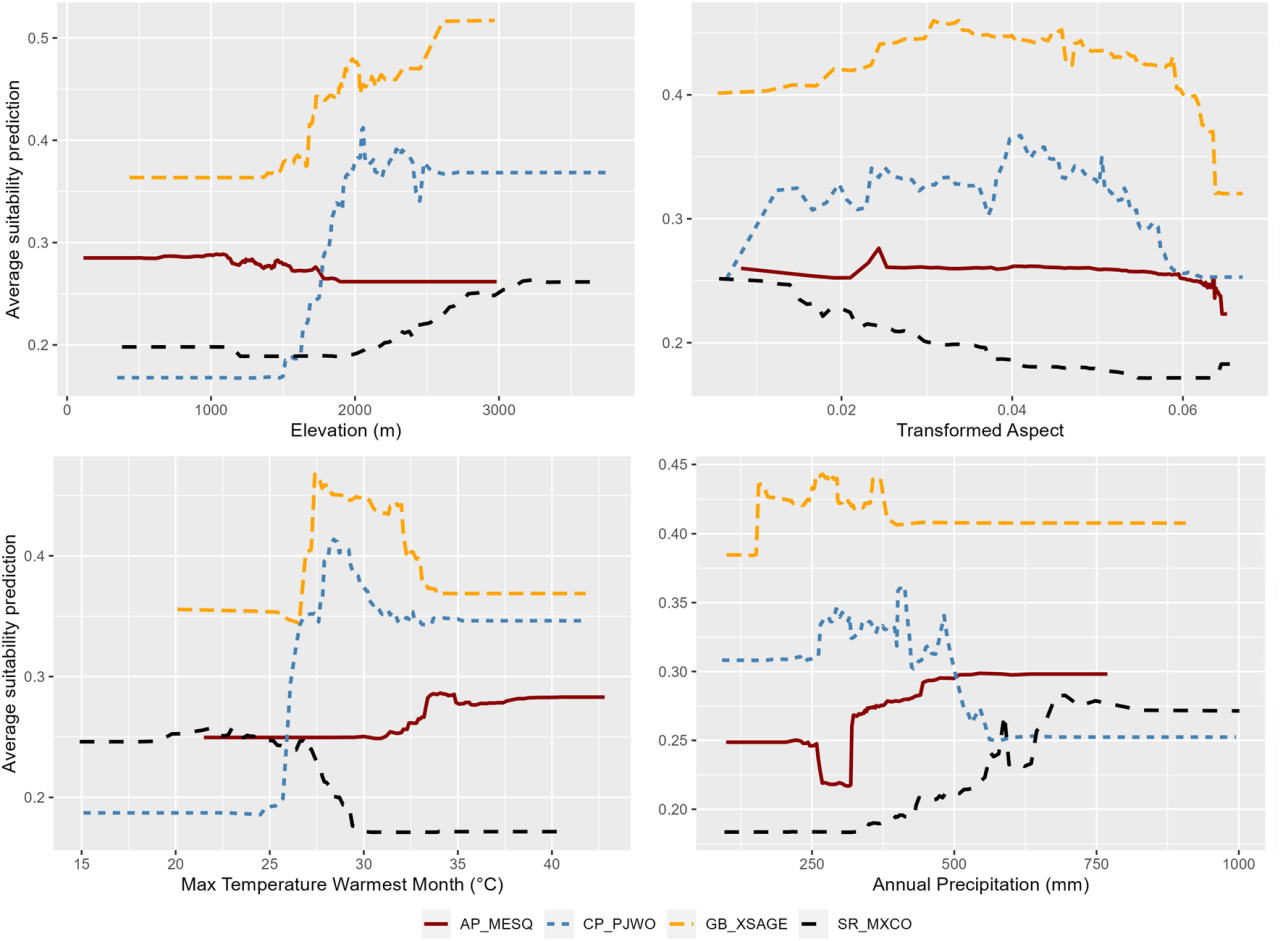
Partial dependence plots illustrating the relationships between elevation, transformed aspect (trasp), maximum temperature of the warmest month (bio5), and annual precipitation (bio12) with predictions of ecosystem suitability for four ecosystems in the southwestern United States (Arizona, Colorado, Nevada, New Mexico, Utah). The ecosystems include: Apacherian-Chihuahuan Mesquite Upland Scrub (AP_MESQ), Colorado Plateau Pinyon Juniper Woodland (CO_PJWO), Great Basin Xeric Mixed Sagebrush Shrubland (GB_XSAGE), Southern Rocky Mountain Dry-Mesic Montane Mixed Conifer Forest and Woodland (SR_MXCO). The vertical axis shows the average prediction of ecosystem suitability based on 100 sample locations. While each of these variables affects the suitability of a given location for the establishment and maintenance of these ecosystems, the relationships between these variables and ecosystems differ.

Ecosystems can have similar variable importance metrics with different relationships between them (Fig. 3). Ecosystem suitability for Colorado Plateau Pinyon Juniper Woodland and Great Basin Xeric Mixed Sagebrush Shrubland, for example, increased sharply at 1500 m elevation. Suitability remained relatively constant for the woodland with elevations ≥ 2000 m, while suitability continues increasing for the shrubland ecosystem up to 3000 m (Fig. 3). For maximum temperature during the warmest month (bio5), ecosystem suitability quickly declined at temperatures > 26 °C for the Southern Rocky Mountain Dry-Mesic Montane Mixed Conifer Forest and Woodland, while suitability for Apacherian-Chihuahuan Mesquite Upland Scrub began increasing at 31 °C (Fig. 3).

**Figure 4:**
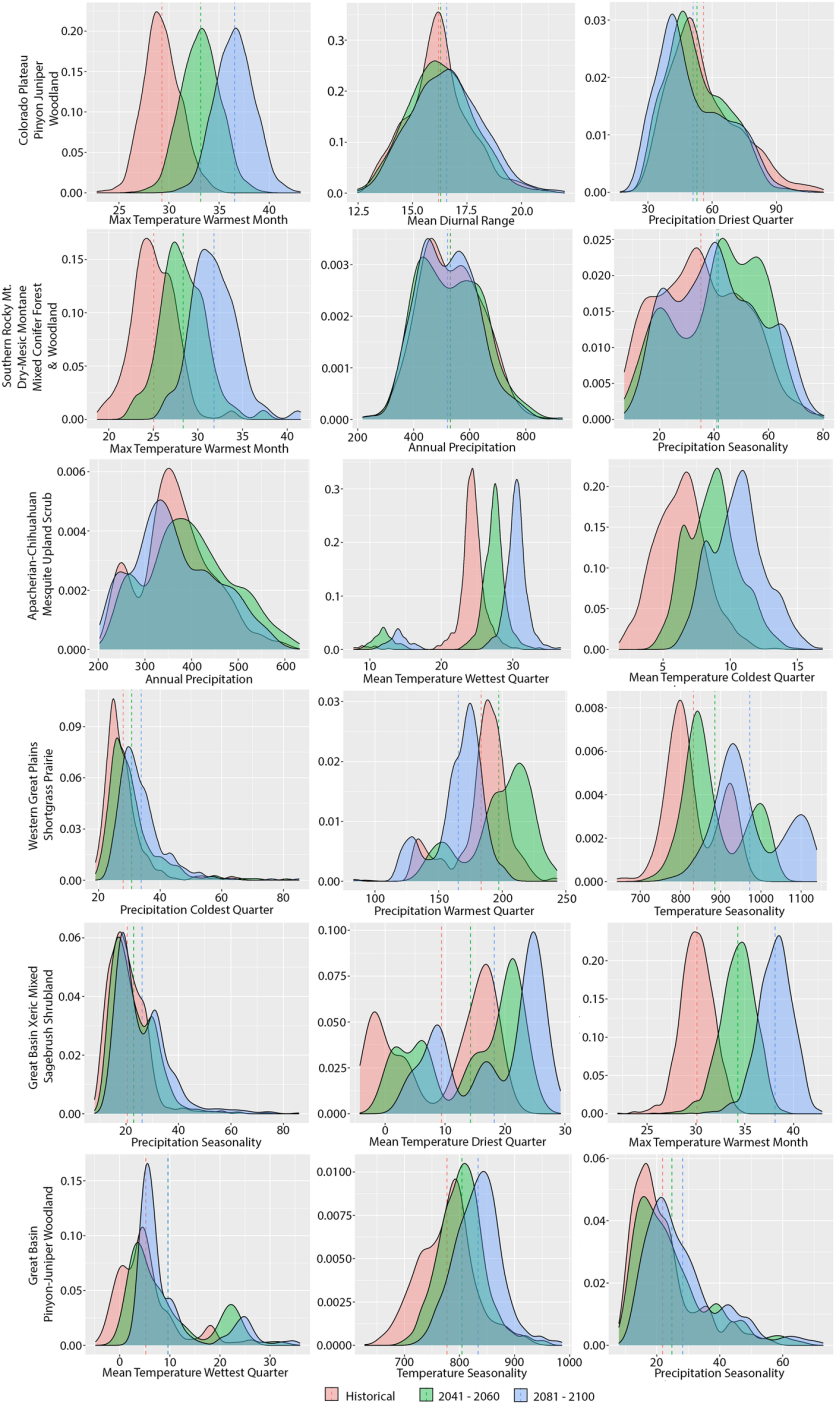
Density plots for the three most supported bioclimatic variables predicting climate suitability for six out of 22 ecosystems within the southwestern United States (Arizona, Colorado, Nevada, New Mexico, Utah). For each ecosystem, these data were sampled from points within their historical range (pink, 1970–2000) and resampled with the projected bioclimatic predictions for the mid-century (blue; 2041–2060) and end-of-century periods (green; 2081–2100), depicted here with the BCC-CSM2-MR climate model and SSP3-7.0 emissions scenario. The vertical axes have different scales for each ecosystem and bioclimate variable. For each of these ecosystems, bioclimates shift within their historical locations, causing changes in ecosystem suitability that affect an ecosystems future establishment, resilience and resistance.

Future combinations of these abiotic variables drive ecosystem suitability. Consequently, we projected changes in the amount and locations of bioclimatic conditions using SSP3-7.0 with CMIP6 BCC-CSM2-MR and GFDL-ESM4 climate models and SSP5-8.5 with BCC-CSM2-MR, to predict future climatic effects on ecosystem suitability within each ecosystems’ historical location (Fig. 4, Supplementary Table 1). For the Colorado Pinyon Juniper Woodlands ecosystem, the historical mean of maximum temperature during the warmest month (bio5) is 29.3 °C (SD 1.8 °C), which increased to 36.5 °C (mean; SD 1.9 °C) by end-of-century (Fig. 4; Supplementary Table 1). At end-of-century, bio5 also rises from a historical mean of 25.0 °C (SD 2.4 °C) to 31.8 °C (SD 2.6 °C) in the Southern Rocky Mountain Dry-Mesic Mixed Conifer Forest and Woodland, and from a historical mean of 29.8 °C (SD 1.8 °C) to 38.1 °C (SD 1.8 °C) in the Great Basin Xeric Mixed Sagebrush Shrubland (Fig. 4; Supplementary Table 1). Precipitation of the warmest quarter (bio18) influences the Western Great Plains Shortgrass Prairie ecosystem (Fig. 2), with the historical mean of 183.4 mm (SD 22.2 mm) projected to 197.2 mm (SD 26.1 mm) by mid-century, which drops to 165.5 mm (SD 19.5 mm) by end-of-century (Fig. 4, Supplementary Table 1).

Given all bioclimate temperature variables, ecosystems, climate projections, emission scenarios, and future periods, 74% of model combinations have temperature variables increasing (*n* = 968 combinations). The following variables were positive and increasing in ≥ 92% of the models: annual mean temperature (bio1), temperature seasonality (bio4), maximum temperature of the warmest month (bio5), mean temperature of the wettest quarter (bio8), mean temperature of the driest quarter (bio9) and mean temperature of the warmest quarter (bio10). Alternatively, minimum temperature during the coldest month (bio6) is typically negative (91% of model combinations).

For all 8 precipitation variables, 64% of the ecosystem, climate model, emission and temporal combinations are positive, indicating projected increases (*n* = 704 combinations). The following variables were typically positive: precipitation seasonality (bio15; 95%), precipitation during the wettest month (bio13; 89%), precipitation during the wettest quarter (bio16; 84%) and precipitation of the warmest quarter (bio18, 82%). The amount of precipitation during the driest month (bio14) and the amount of precipitation during the driest quarter (bio17) were typically negative across ecosystems, indicating seasonal drying conditions (92% and 85% of model combinations, respectively).

**Figure 5:**
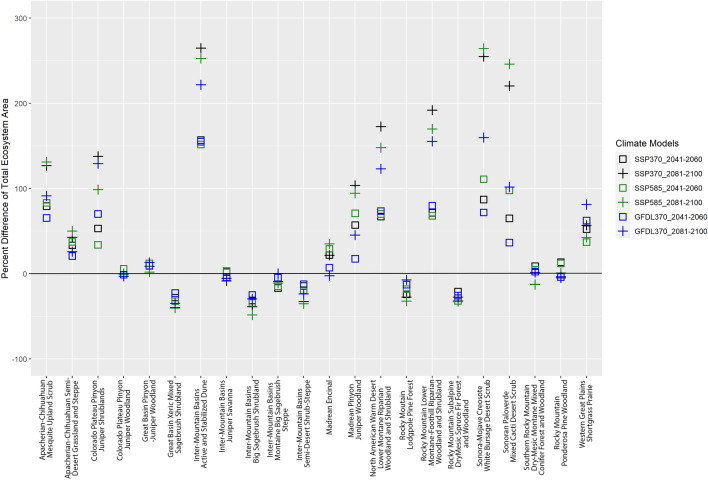
Percent difference in total area of ecosystem suitability for 22 ecosystems within the southwestern United States (Arizona, Colorado, Nevada, New Mexico, Utah). Percent difference calculations are based on the differences in the area between the historic baseline and the area predicted from climate models (BCC-CSM2-MR SSP3-7.0 (SSP370, black), BCC-CSM2-MR SSP5-8.5 (SSP585, green), GFDL-ESM4 SSP3-7.0 (GFDL370, blue)) occurring at mid-century (2041–2060; represented by squares) and end-of-century (2081–2100; represented with a “ + ”). Area is calculated as the proportion of ecosystem suitability within a pixel multiplied by pixel area (16.7 km^2^). These calculations consider pixels with suitability values ≥ 0.33 (except for the Inter-Mountain Basins Juniper Savanna ecosystem which included values ≥ 0.16).

Ecosystem suitability is changing within an ecosystems’ historical range in concert with the magnitude and direction of bioclimatic shifts occurring within that historical range. Likewise, shifts in bioclimate over time and geography raise ecosystem suitability outside an ecosystems traditional range, enabling its establishment in novel locations. One measure of these effects is the amount and changes in total area of ecosystem suitability over time (Fig. 5). By mid-century, for 16 ecosystems (BCC-CSM2-MR with SSP3-7.0 and SSP5-8.5 scenarios) and 13 ecosystems (GFDL-ESM4 and SSP3-7.0), the average percent increase in the amount of suitable area for each ecosystem rises by ~ 50% (~ 40% SD). By end-of-century, the mean percent increase in area for 13 ecosystems is ~ 120% (BCC-CSM2-MR SSP3-7.0, SSP5-8.5; SD ~ 93%) or ~ 90% (GFDL-ESM4 SSP3-7.0; SD ~ 69%). The 6 ecosystems decreasing in suitable area (BCC-CSM2-MR SSP3-7.0, SSP5-8.5 scenarios) lost an average of ~ 25% by mid-century (SD ~ 7%) with 9 ecosystems within the GFDL-ESM4 scenario predicted to lose 12% (SD 10%; SSP3-7.0; Fig. 5). By end-of-century, for ecosystems experiencing suitability decline, the amount of suitable area lost remains comparable to mid-century values.

The Sonora-Mojave Creosote-White Bursage Desert Scrub ecosystem had considerable increases in suitable area, doubling by mid-century and tripling by end-of-century. Ecosystems losing the most suitable area included the Inter-Mountain Basins Big Sagebrush Shrubland, Great Basin Xeric Mixed Sagebrush Shrubland, and Rocky Mountain Subalpine Dry Mesic Spruce Fir Forest and Woodland. Collectively, their range of decreasing area is ~ 20–30% by mid-century and 30–50% at end-of-century (Fig. 5). Across all climate change projections, 5 ecosystems displayed at least one instance of percentage changes in area increasing by mid-century and then decreasing by end-of-century (e.g., Southern Rocky Mountain Dry-Mesic Montane Mixed Conifer Forest and Woodland and the Rocky Mountain Ponderosa Pine Woodland; Fig. 5).

Suitability for 18–20 of these ecosystems increased in elevation 12–15% by mid-century and 17–25% by end-of-century (scenario dependent). The Sonora-Mojave Creosote White Bursage Desert Scrub ecosystem had a 40–65% elevation increase by mid-century and 100–140% by end-of-century (Supplementary Fig. 1). For this ecosystem, elevation increases from a historical mean of ~ 550 to 800 m (mean) by mid-century and a 1200 m (mean) by end-of-century. Mean elevational changes in suitability for the Chihuahuan Loamy Plains Desert Grassland increase from 1500 m historically to 1800 m at mid-century and 1900 m by end-of-century.

Projections indicate most ecosystems migrating northward (*n* = 15–18 (depending on emission scenario); Supplementary Fig. 2). On average, increases in northing are 4–6% at mid-century and 8% at end-of-century. The Apacherian-Chihuahuan Mesquite Upland Scrub ecosystem, for instance, has a historical northing of ~ 1,180,000 m (mean) that increased 13% by mid-century (1,328,000 m) and 24% by end-of-century (1,470,000 m; BCC-CSM2-MR SSP3-7.0). Latitudinally, the historical northing of Truth or Consequences, New Mexico shifts to Santa Fe, New Mexico by end-of-century.

**Figure 6:**
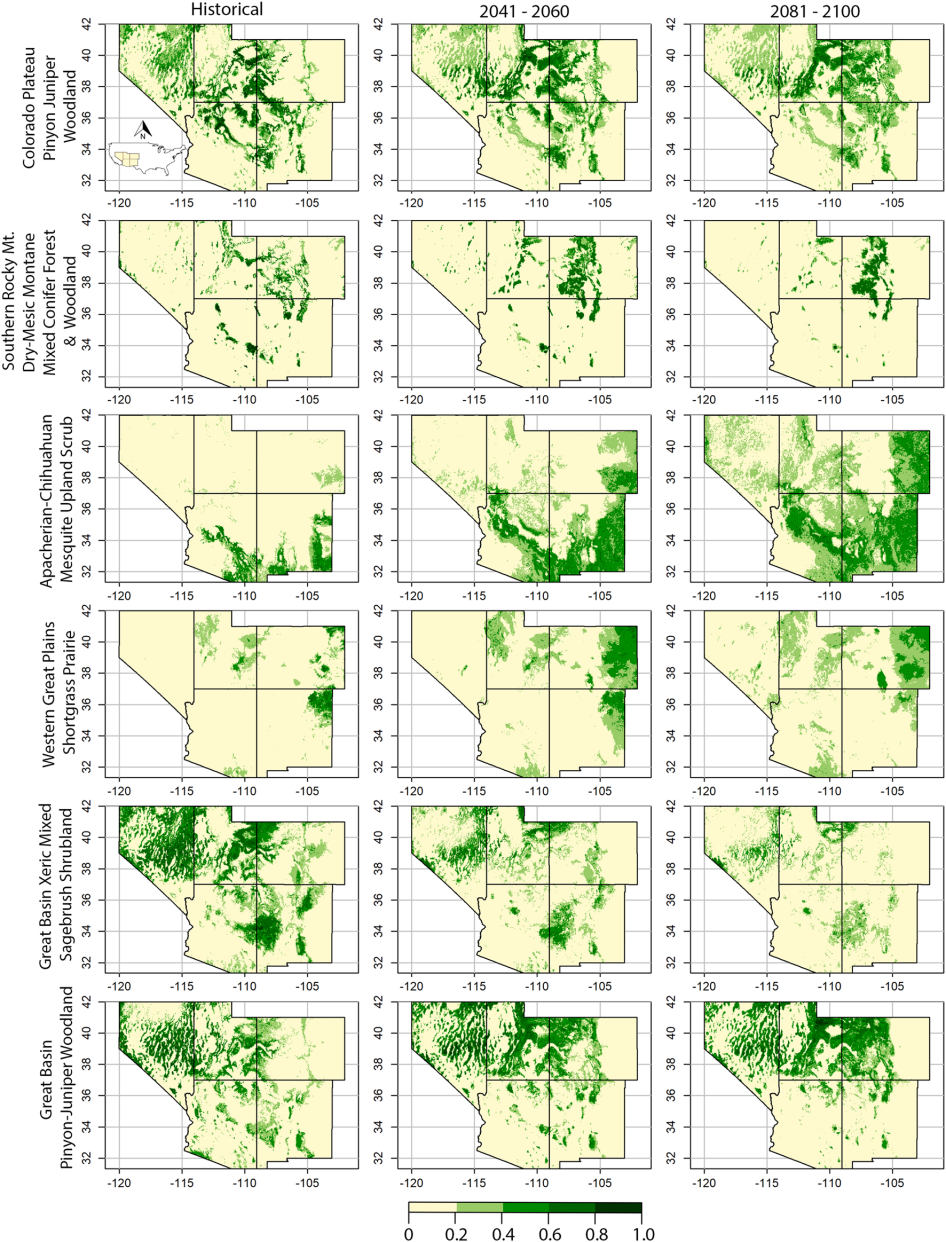
Historical (1970–2000), mid-century (2041–2060) and end-of-century (2081–2100) predictions of climate suitability for 6 of 22 ecosystems located in the southwestern United States (inset; states clockwise from upper left: Nevada, Utah, Colorado, New Mexico, Arizona). The suitability predictions are based on relationships between 19 downscaled bioclimatic variables and three terrain variables occurring within the historical locations for each ecosystem. These relationships identify the combinations of bioclimatic and terrain variables that best predict an ecosystem’s suitability (16.7 km^2^ pixel resolution) for establishing and maintaining an ecosystem. Mid-century and end-of-century spatial predictions of ecosystem suitability are based on climatic models and emission scenarios (BCC-CSM2-MR SSP3-7.0 exemplified here) that project future states of bioclimate variables. Colors indicate the predicted ecosystem suitability, ranging from pale yellow to dark green, based on each ecosystem’s relationship with abiotic variables within the historic and future locations (continuous pixel values spanning 0.0 – 1.0; R version 4.2.3 https://www.r-project.org/).

For land stewards, geospatial data identifying where and when shifts in ecosystem suitability are projected to occur directly informs their on-the-ground mitigation for future ecosystem change. As examples, suitability for the Great Basin Pinyon Juniper Woodland increased at northern latitudes while receding in the southern latitudes by end-of-century (Figs. 1 and 6). Ecosystem suitability for the Southern Rocky Mountain Dry-Mesic Montane Mixed Conifer Forest and Woodland decreases in Arizona and New Mexico while rising in Colorado and Utah (Figs. 1 and 6).

**Figure 7:**
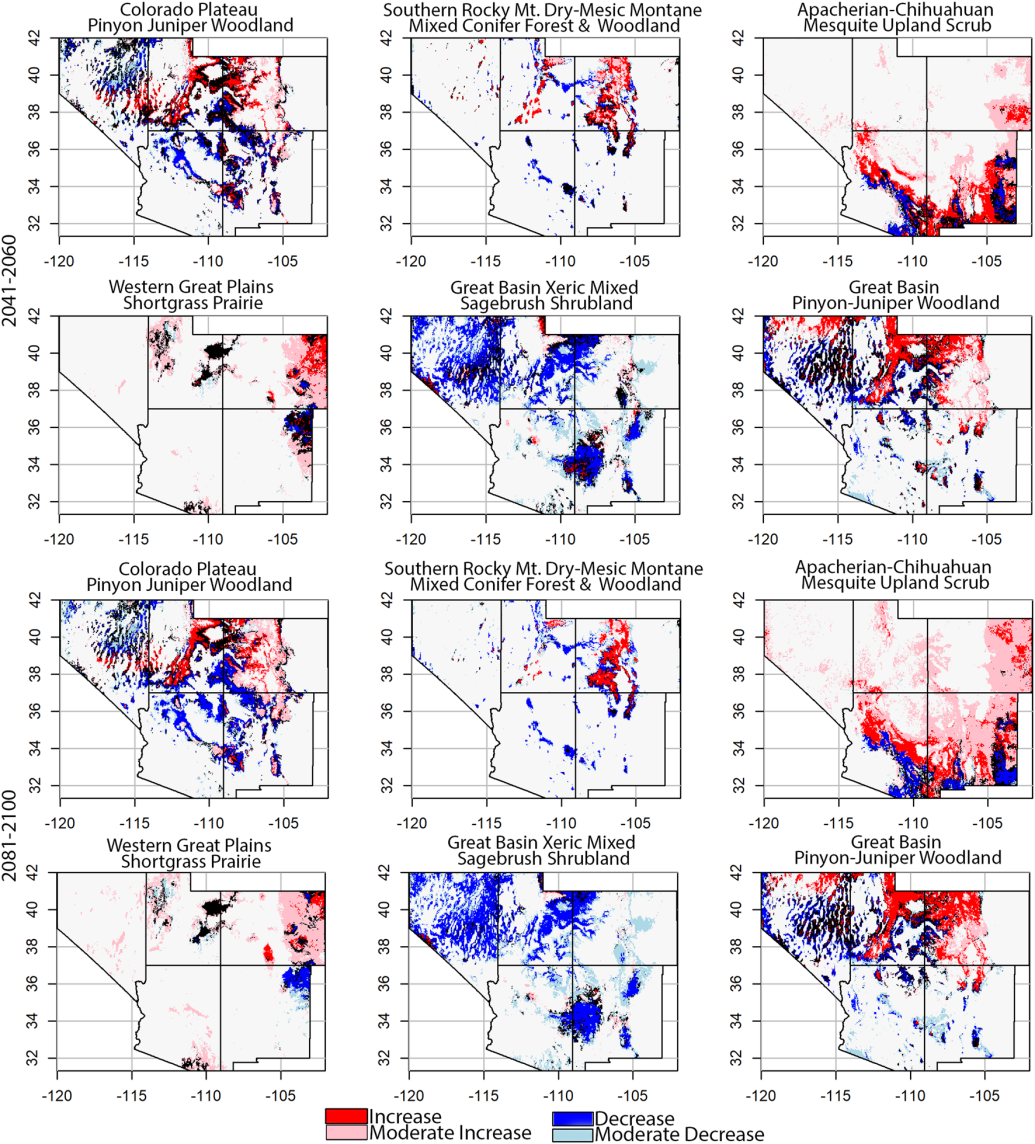
Spatially-explicit changes in ecosystem suitability for 6 ecosystems within the southwestern United States, from historical (1970—2000) to mid-century (2041–2060) and end-of-century (2081–2100) periods, based on projected bioclimate from the BCC-CSM2-MR SSP3-7.0 climate model and emission scenario (Arizona, Colorado, New Mexico, Nevada, Utah). Red indicates locations with mid-century suitability ≥ 0.5 and historic suitability < 0.5 (high increase in ecosystem suitability). Pink shows locations with mid-century suitability ≥ 0.25 and < 0.5 with historic suitability < 0.25 (moderate increase in ecosystem suitability). Blue identifies locations with mid-century suitability < 0.5 and historic suitability ≥ 0.5 (large loss in ecosystem suitability). Light blue identifies locations with mid-century suitability < 0.25 and historic suitability ≥ 0.25 and < 0.5 (moderate loss in ecosystem suitability). Black colors locations with suitability unchanged.

Examination of ecosystem suitability values provides a greater perspective on these geographical shifts (Fig. 7). For instance, the historical distribution for Apacherian-Chihuahuan Mesquite Upland Scrub (157,000 km^2^) rose 80% by mid-century (Fig. 5), with 74% of the suitable area increasing (locations having historical suitability < 0.5 increasing to ≥ 0.5, and areas with suitability values < 0.25 increasing to ≥ 0.25 and < 0.5 by mid-century (Fig. 7)). By end-of-century, suitable area rose to 357,000 km^2^, with 82% of the area having suitability higher than historical levels (Figs. 5 and 7). Most suitability decreases occurred in the southern latitudes, with suitability shifting upslope and northward (Fig. 7; Supplementary Fig. 1, Supplementary Fig. 2). Conversely, the Great Basin Xeric Mixed Sagebrush Shrubland is predicted to decline 28% in suitable area by mid-century, with 66% of the area in suitability loss (Figs. 5 and 7). At end-of-century, 0.5% of the area has suitability increases ≥ 0.5 that historically were < 0.5, with 86% of total area in suitability decline (Figs. 5, 6 and 7).

**Figure 8:**
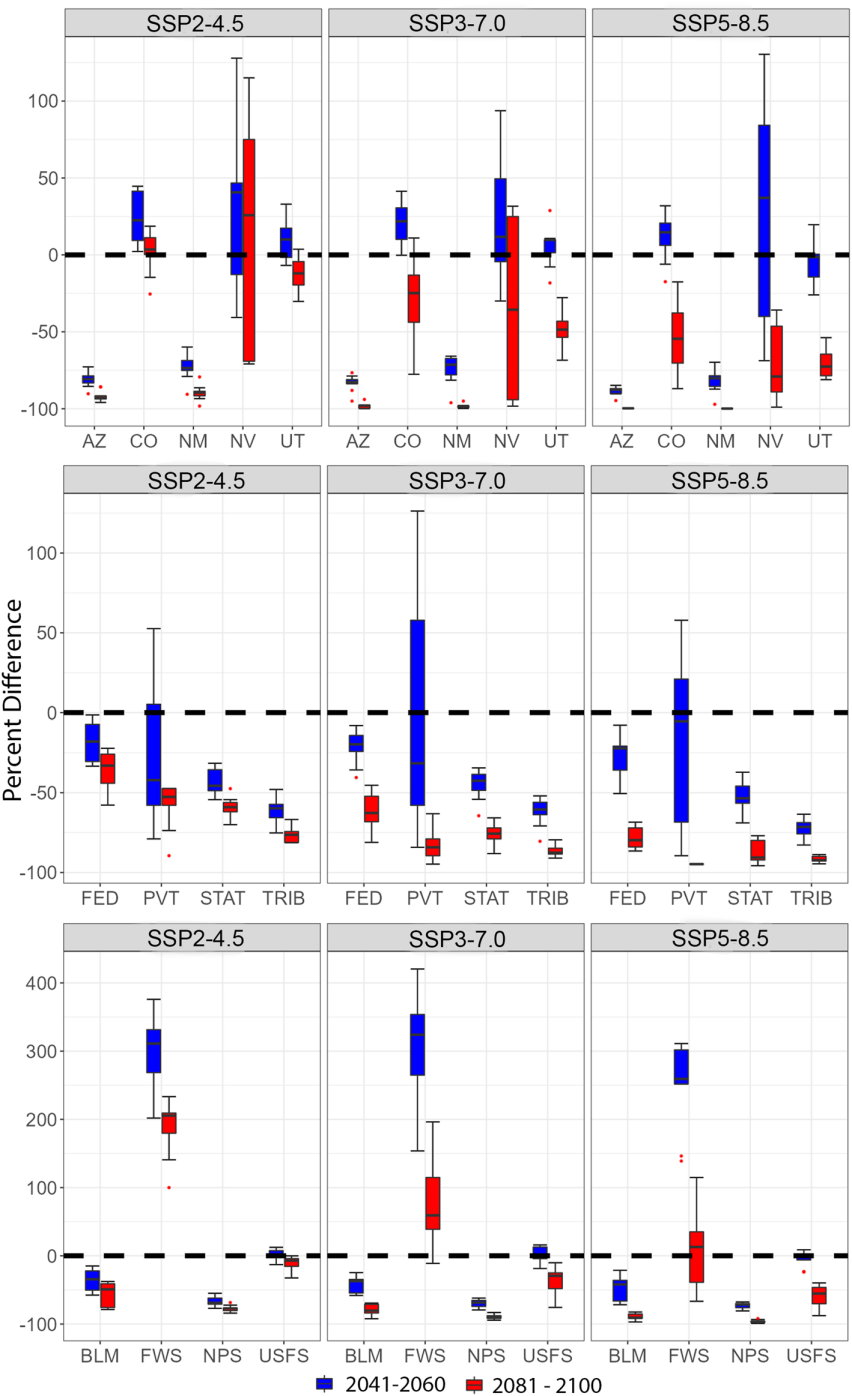
Box plots describing the percent difference in the amount of suitable area for the Colorado Plateau Pinon Juniper Woodland ecosystem between the historical, mid-century (2014–2060; blue) and end-of-century (2081–2100; red) periods, based on ensemble model results from 9 GCMs (suitability ≥ 0.5, Supplementary Table 3). These GCMs span three shared socioeconomic pathways varying from low to high greenhouse gas emissions scenarios and levels of warming (SSP2-4.5, SSP3-7.0, SSP5-8.5). The top panel parses percent changes in area by state (Arizona (AZ), Colorado (CO), New Mexico (NM), Nevada (NV), Utah (UT)) the middle panel by ownership (Federal (FED), Private (PVT), State (STAT) and Tribal (TRIB)), and the bottom panel by Federal ownership (Bureau of Land Management (BLM), United States Fish and Wildlife Service (FWS), National Park Service (NPS), and United States Forest Service (USFWS); R version 4.2.3 https://www.r-project.org/).

Changes in ecosystem suitability showed strong geographic variation by state (i.e., latitude) and land jurisdiction (Fig. 8). In Arizona and New Mexico, the Colorado Plateau Pinon Juniper Woodland ecosystem loses suitability (> 0.5) with a ~ 75% decline predicted by mid-century and nearly 100% loss at end-of-century, with gains in Colorado and Nevada (mean of 9 climate models with SSP3-7.0 and SSP5-8.5; Fig. 8). Suitable area declined > 50% across jurisdictions, with Tribal land losing the greatest proportion (Fig. 8). Within Federal jurisdictions, the total area remains unchanged within USDA Forest Service property at mid-century, but declines ~ 50% by end-of-century (Fig. 8). The Bureau of Land Management (BLM) loses ~ 25% of highly suitable area by mid-century and 50–75% by end-of-century (depending on emission scenario; Fig. 8). When predictions of ecosystem suitability in state and land ownerships have less uncertainty, it indicates greater confidence in the amount of ecosystem suitability within them (Fig. 8; Supplementary Fig. 3). Indeed, variability tends to be lower in New Mexico and Arizona, adding further support to future declines in ecosystem suitability for Colorado Plateau Pinon Juniper Woodlands in these states (Fig. 8; Supplementary Fig. 3).

Results from all properties (*n* = 52,565) reveal changes in ecosystem suitability of different magnitudes and types. As examples, we present spatial depictions of ecosystem suitability values describing the conditions required for establishing and maintaining ecosystems during the historical (1970–2000) and mid-century periods (2041–2060) with the BCC-CSM2-MR SSP5-8.5 climate model and emission scenario (Fig. 9). San Andres National Wildlife Refuge within southern New Mexico (232 km^2^), for example, contained 26 km^2^ of historical, suitable area for Apacherian-Chihuahuan Mesquite Upland Scrub (Fig. 9). By mid-century, suitable area expands to 92 km^2^ (SSP5-8.5; Fig. 9). The amount of annual precipitation (bio12) influences this ecosystems’ suitability (Figs. 2, 9). Historically, the amount of annual precipitation on San Andres NWR spanned 250–495 mm (location dependent, Fig. 9). At mid-century, projections indicate most of the Refuge experiencing annual precipitation between 290 and 370 mm, generating conditions more favorable to this ecosystem type (BCC-CSM2-MR SSP5-8.5; Fig. 9). Tonto National Forest (11,600 km^2^) occurs in southcentral Arizona and historically contained 1705 km^2^ of area suitable for Rocky Mountain Ponderosa Pine Woodland (Fig. 9). By mid-century, suitable area halves under all emission scenarios and declines 60% at end-of-century (Fig. 9). The maximum temperature of the warmest month (bio5) is a dominant predictor of this ecosystem (Fig. 9). Forest-wide, conditions become unfavorable for sustaining Ponderosa pine woodlands as the average historical temperature maximum of 34.7 °C (SD 3.1), increases to 38.7 °C by mid-century (SD 3.2) and 41.5 °C (SD 3.2; BCC-CSM2-MR SSP5-8.5) by end-of-century (Fig. 9). Rocky Mountain National Park (~ 1000 km^2^), situated in northern Colorado, historically contained 46 km^2^ of area suitable for the Southern Rocky Mountain Dry-Mesic Montane Mixed Conifer Forest and Woodland (Fig. 9). This amount rises to ~ 450 km^2^ by mid-century and ~ 500–600 km^2^ by end-of-century (Fig. 9). The amount of annual precipitation (bio12) is a strong predictor of suitability (Figs. 2, 9). While the average amount of annual precipitation within the park remains similar between periods (historical 677.6 mm (108.8 SD); mid-century 650.2 (68.8 SD); Fig. 9), the eastern and southwestern borders of the park are predicted to have increased precipitation, favoring this ecosystems’ suitability.

**Figure:**
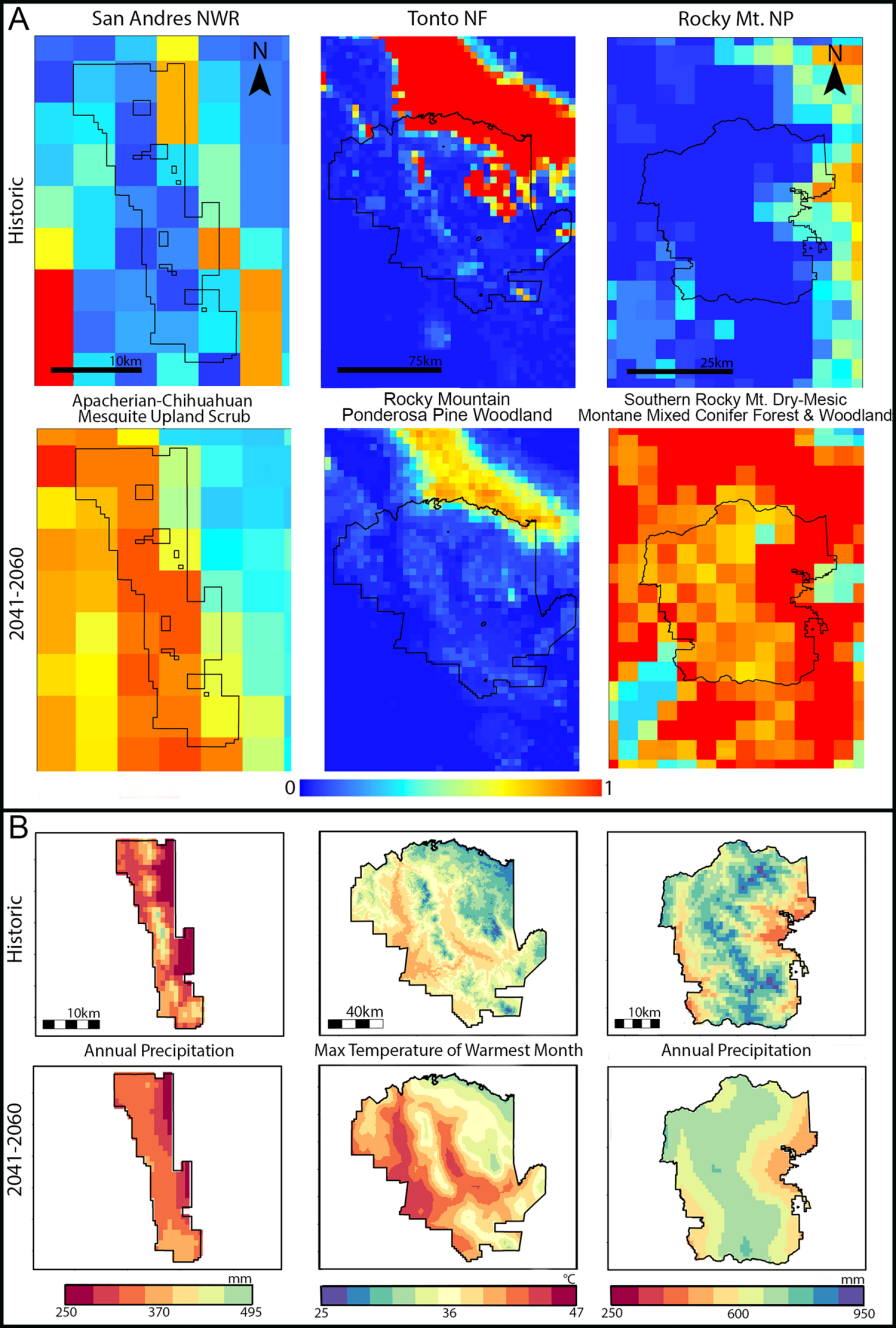
Spatial depictions of ecosystem suitability values (panel A), describing the conditions required for establishing and maintaining ecosystems (16.7 km^2^ pixel) during the historical (1970–2000) and mid-century periods (2041–2060) with the BCC-CSM2-MR SSP5-8.5 climate model and emission scenario. Red indicates pixels with high suitability (values = 1.0) and dark blue colors pixels having low suitability (values = 0.0). The plots in panel B depict bioclimate variables having high influence in predicting the suitability of that ecosystem. Plots on the far left (**A**) indicate the predicted suitability for the Apacherian-Chihuahuan Mesquite Upland Scrub ecosystem in the San Andres National Wildlife Refuge (NWR), which increases over time, partly due to the decline in annual precipitation (**B**). The middle plots (**A**) show how ecosystem suitability for the Rocky Mountain Ponderosa Pine Woodland in the Tonto National Forest (NF) declines by mid-century, as the maximum temperature of the warmest quarter increases (**B**). Plots on the right (**A**) depict ecosystem suitability for the Southern Rocky Mountain Dry-Mesic Montane Mixed Conifer Forest and Woodland in Rocky Mountain National Park, which increases over time due to rising annual precipitation on the western and eastern borders of the park ((**B**) R version 4.2.3 https://www.r-project.org/).

## Discussion

Land-management agencies experience ecosystem shifts and compositional transformations occurring throughout their jurisdictions, and recognize the range of response options available, like resisting (i.e., forest thinning to reduce soil moisture demand and tree canopy fuels), accepting, or directing climate induced changes (e.g., species relocations outside historical ranges; seeding burned areas with a mixture of historical and transitional species)^7,8^. These agencies want deliberative mitigation strategies, but lack foundational data describing the relationships between climate change and ecosystems, along with maps predicting ecosystem suitability in future periods, at scales relevant for informing them.

Our project offers a procedure that supplies these data for addressing the effects of climate change at the scales land managers work. We built relationships between abiotic variables and the historical, geographical locations for southwestern ecosystems to identify the most influential abiotic variables for predicting the suitability of an area to establish and maintain these ecosystems. Model predictions were tested with independent validation data, with most (17) displaying robust performance (Table 1). Ecosystems limited by sample size had lower scores for the Sørensen similarity index, and although these models have lower performances and higher predictive uncertainty, they do provide insight into how ecosystem suitability may change by time and location (Table 1).

Over time, climate change alters the bioclimatic variables important for maintaining ecosystem suitability within each ecosystems’ historical boundary. This outcome weakens the sustainability of that historical location to continue supporting the establishment and maintenance of a given ecosystem. Bioclimatic variables are changing outside an ecosystems’ historical range too, generating novel locations suitable for ecosystem establishment or community reassembly, thereby enabling ecosystem shifts outside traditional ranges (Figs. 1, 6 and 7). We found that most predictions of ecosystem suitability associated with temperature increases, while precipitation variables had greater inconsistency (Supplementary Table 1, Supplementary Fig. 4). In general, arid lands are vulnerable to swings in annual and seasonal precipitation cycles and our results resemble this pattern^26^.

We quantified suitability shifts by area, elevation, latitude, and distribution to reveal “how” ecosystems respond to climate change. Most ecosystems increased in suitable area by end-of-century, with suitability for eight ecosystems doubling (Fig. 5). Of the 6 ecosystems decreasing in distribution, they are not projected to lose ≥ 50% of total, historically suitable area (BCC-CSM2-MR SSP3-7.0 and SSP5-8.5; Fig. 5). Even small proportions of suitability loss equate to considerable area for large ecosystems like the Inter-Mountain Basins Montane Big Sagebrush Steppe (111,533 km^2^), predicted to lose 9% (10,168 km^2^) at end-of-century (BCC-CSM2-MR SSP3-7.0, Fig. 5).

Most southwestern ecosystems increased in elevation and latitudinal gradients (Supplementary Fig. 2). These results, combined with the distribution of suitability values within an ecosystem, diagnoses the effects of climate change upon it (Fig. 7). As examples, within the Rocky Mountain Subalpine Dry-Mesic Spruce-Fir Forest and Woodland, the area in high suitability (≥ 0.5) declined 30% by midcentury (BCC-CSM2-MR SSP3-7.0). Predictions for this high-altitude ecosystem include shifts to even higher elevations, which mostly occur at lower latitudes (i.e., southern Colorado, hence the decrease in northing), while abiotic conditions decline elsewhere, thereby reducing the total amount of suitable area (Figs. 1, 5; Supplementary Fig. 1, Supplementary Fig. 2). The Colorado Plateau Pinyon Juniper Woodland ecosystem is predicted to change 0.2% between its historical and end-of-century total area (BCC-CSM2-MR SSP3-7.0; Fig. 5), but the most suitable area (≥ 0.6) decreases 51% (Fig. 7), an amount offset quantitatively but not qualitatively by increases in areas having low suitability, typically at higher elevations and latitudes (Fig. 7, Supplementary Fig. 1, Supplementary Fig. 2).

Since managing ecosystems requires understanding “where” and “when” shifts occur, we predicted, mapped and summarized ecosystem suitability values within 52,565 properties (Figs. 1, 6, 7, 8 and 9, Supplementary Table 2). By mid-century, every property experienced changes in ecosystem suitability. Federal, State, Tribal, and Private land jurisdictions of all sizes (≥ 16.7 km^2^ minimum area), emission pathways, and periods were predicted to experience changes in suitable area ≥ 50% for at least one ecosystem within it (Supplementary Table 2). At San Andres NWR, suitable area of Apacherian-Chihuahuan Mesquite Upland Scrub quadruples by mid-century, influenced by declining annual precipitation, as the amount of suitable area for other ecosystems, like the Apacherian-Chihuahuan Semi-Desert Grassland and Steppe and the Inter-Mountain Basins Semi-Desert Shrub-Steppe halved (Fig. 9; Supplementary Table 2). Tonto National Forest loses much Rocky Mountain Ponderosa Pine Woodland given increases in the maximum temperature during the warmest month, as suitable area for Sonoran Paloverde Mixed Cacti Desert Scrub increases 1.6 times by mid-century (Fig. 9; Supplementary Table 2). These temperature increases likely impact water availability, despite annual precipitation remaining relatively unchanged^27^. Projections for Rocky Mountain National Park indicate widespread increases in suitability for the Southern Rocky Mountain Dry-Mesic Montane Mixed Conifer Forest and Woodland and 10% decreases in Intermountain Sage Steppe by mid-century (Fig. 9; Supplementary Table 2). Ecosystem suitability within all jurisdictions can be similarly examined.

We selected a few informative abiotic variables for each of the ecosystems and jurisdictions, to exemplify and help simplify visualizations of why and where temporal and geographical changes in ecosystem suitability occur. Properties containing less information (i.e., fewer pixels) for examining spatial and temporal changes in ecosystem suitability, would benefit by taking a larger landscape perspective. Analyses could incorporate changes in ecosystem suitability within and around a focal property, to subsequently deduce and interpret projected changes occurring inside it. Importantly, the amount of ecosystem suitability occurring at a given location within or outside these jurisdictions relies on the interplay among all abiotic variables as determined historically (Fig. 2), plus the GCMs and emission scenarios selected to make future projections. Plots describing the mean and variability in the amounts of ecosystem suitability across geographical space and time show where projected values are more consistent or uncertain (Fig. 8, Supplementary Fig. 3).

The dominant species within each ecosystem influence the bioclimatic and ecosystem relationships^6,28^. When ecosystems share dominant species, predictions of ecosystem suitability can overlap, even when variable importance differs (Supplementary Table 1). This situation occurs in the Colorado Plateau Pinyon Juniper Woodland, Great Basin Pinyon-Juniper Woodland and Madrean Pinyon Juniper Woodland. These ecosystems are biogeographically based, so if one is working on pinyon-juniper habitats in New Mexico, the ecosystem would be classified as Colorado Plateau Pinyon Juniper Woodland. Were one particularly concerned about this mixing effect, the spatial predictions for the pinyon-juniper ecosystems could simply be combined.

Ecosystems in geographical locations with declining climatic suitability can persist and resist changes until they experience a pronounced ecological event like extreme fire, prolonged drought, or insect outbreaks^13,29^. Afterwards, the ecosystem may lose resilience, struggle to reestablish, and be supplanted with a different ecosystem better suited to the new abiotic regime^29–31^. The bioclimatic conditions influencing ecosystem suitability may also shift faster than ecosystems can track, causing composition changes and transitions to novel ecosystem states^14,32,33^. Observations on post-disturbance ecosystem recovery or controlled experiments help reveal such changes.

Our approach relates abiotic data from the recent past with ecosystem presence, to project ecosystem suitability in the future. Albeit a common approach for ecological modeling, we recognize that the historical composition of ecosystems is also influenced by other factors besides bioclimate (e.g., depopulation in Native Americans, long-term atmospheric features, fire^34,35^).

Our procedures for quantifying and summarizing ecosystem suitability applies to any region, given ecosystem locations (field sampled or remotely sensed) and downscaled bioclimatic data. Results predict ecosystem suitability, the conditions required for maintaining and establishing ecosystems. Temporal and spatial suitability changes will cause ecosystem replacement or shifts to novel locations, with some ecosystems moving intact and others in pieces^14,36^.

All properties we examined were predicted to experience ecosystem shifts. The response of the animals and plants within them depends on the species vagility, landscape connectivity, characteristics of the property and neighboring land uses (e.g., agriculture, urban development, transportation corridors). Some species will track ecosystems shifts, while others require human intervention. Assisted migration is one example of a mitigation approach for moving species from historical ranges into novel locations^37^. Our data informs assisted migration, by identifying where and when ecosystem suitability declines, thereby threatening focal species, and predicting alternative areas suitable for species introduction. Working collaboratively, some properties could resist ecosystem shifts, buying time as other properties accept (or direct) ecosystem shifts, thereby providing suitable areas to pursue natural or assisted migration^37^.

In practice, projections of ecosystem suitability should incorporate GCMs and emission scenarios chosen by the partners, so the assumptions in model inputs align with their requirements and produce results they want for building mitigation strategies. We chose two GCMs and emission scenarios to design the method, demonstrate the process, and exemplify the utility of results to inform landscape-scale mitigation for climate change. Our approach can incorporate any number of GCMs and emission scenarios.

Best scientific practices dictate that mitigation strategies be informed by a suite of GCMs, to quantify uncertainty in model predictions. Therefore, we used 9 GCMs with three emission scenarios to predict ecosystem suitability within the Colorado Plateau Pinyon Juniper Woodland and exemplify the importance and relevance of integrating uncertainty into mitigation design (Fig. 8; Supplementary Fig. 3, Supplementary Table 3). Geographical locations having low predictive uncertainty indicate places with greater confidence in the suitability predictions (Fig. 8, Supplementary Fig. 3). For this ecosystem, the southern latitudes (e.g., Arizona and New Mexico) are likely to lose > 50% suitable area (from historical levels), given the low amounts of prediction uncertainty (Fig. 8, Supplementary Fig. 3). Higher latitudes (Colorado) display more uncertainty, with consistent gains in ecosystem suitability at mid-century, followed by suitability loss at end-of-century (Fig. 8, Supplementary Fig. 3). In southern latitudes, were this ecosystem to suffer severe disturbance (e.g., drought, disease, fire) the ecosystem is unlikely to return to its prior state (loses resilience). Likewise, the National Park Service (NPS) is likely to lose much ecosystem suitability for Colorado Plateau Pinyon Juniper Woodland (low uncertainty), while projections for the US Fish and Wildlife Service have higher uncertainty with suitability declines by end-of-century (Fig. 8). Changes in the suitability for this ecosystem are already discernable, exemplified by increases in tree mortality rates and transitioning plant distributions caused by drought and insect outbreaks^38,39^.

The appropriate mitigation response depends on the climatic context. Mitigation responses should be proportional to the risks of potential changes in ecosystem suitability. In this example, many locations in Arizona and New Mexico are projected to experience climatic regimes removed from those suitable for the maintenance and establishment of Colorado Pinyon Juniper Woodland, while locations in Colorado display less climatic divergence (Figs. 6, 7 and 8). Properties in Arizona and New Mexico may focus on accepting ecosystem shifts, and transition to open grass and shrublands, while locations in Colorado practice resistance to mitigate suitability loss, as ecosystem suitability shifts northward (Figs. 6, 7 and 8, Supplementary Fig. 2). Similarly, if conserving Colorado Pinyon Juniper Woodland is important to the NPS, they can work to manage new properties where this habitat remains stable, or partner with other land managers to ensure stewardship of this ecosystem elsewhere. Integration of data describing the relationships between ecosystems and bioclimate, suitability predictions, and the proactive monitoring of bioclimatic conditions help agencies assess the status and trends in ecosystem suitability within their properties to inform such mitigation strategies.

Our results should alert agencies to the scope and scale of ecosystem changes affecting their properties spanning regional geographies. As climate change affects ecosystems regionally, addressing this issue requires collective action matching the regional scale. Land stewards (e.g., Federal, State, Tribal, Private) must build comprehensive and coordinated implementation strategies to generate solutions having greater chances of success. Properties acting independently risk haphazard, ineffectual responses (i.e., imagine a property accepting changes while its neighbor resists them). Collaboration at such scales rarely occurs, and irrespective of the ecological obstacles, the social and institutional challenges are formidable. Few precedents exist, with success hinging on leveraging shared concerns about resource degradation and sustainability, making accomplishments early in the process and participants’ long-term commitment^40–44^. Understanding the nature and timing of ecosystem shifts at regional scales permit such systematic, jurisdictional integration, to address the effects of climate change on ecosystems and the species inhabiting them.

## Methods

We modeled and mapped ecosystem suitability in the states of Arizona, Colorado, New Mexico, Nevada and Utah within the southwestern continental United States (1,390,512 km^2^; Fig. 1). We established relationships between these ecosystems and 19 bioclimate variables from downscaled historical WorldClim v2.1 2.5-min grids (https://www.worldclim.org/) representing historical and future GCM projections^45^. We included terrain information, represented as slope degree, elevation, and transformed aspect (which measures potential solar radiation). These terrain variables were developed using a 90 m digital elevation model (DEM) from the shuttle research and topography mission (SRTM, Table 2). All raster data were scaled to the 2.5-min bioclimate layers using nearest neighbor resampling in the raster package v. 3.5–21 for R statistical software (3.6 × 4.6 km: 16.7 km^2^ pixel)^46^.

Spatially referenced field data for each ecosystem consisted of 10 m × 10 m and 20 m × 20 m plots with percent cover by plant species collected between 2000 and 2003 by the USGS National GAP Analysis Program^47^. We extracted plots and ecosystem occurrence records for each state using ecosystem classifications assigned to plots according to NatureServe and the International Vegetation Classification (IVC) system at the macrogroup level [i.e., vegetation type defined by diagnostic plant species and growth forms^48^. Data queries and processing were completed using the RODBC v. 1.3–19^49^ and rgdal v. 1.5–32^50^ for R statistical software v. 4.2.1. We selected ecosystems with a minimum of 50 occurrence records. The average number of independent occurrence records (e.g., occurring within a single grid cell) was 742 (SD 851) per ecosystem (Table 1).

We modeled ecosystem suitability by intersecting ecosystem plots with the bioclimate grids, representing average historical climate conditions between 1970 and 2000^45^. We used the CMIP6 BCC-CSM2-MR and GFDL-ESM4 GCMs, representing moderate climate sensitivity (the potential warming based on a doubling of atmospheric CO_2_ concentrations) for all 22 ecosystems. We included the SSP3-7.0 and SSP5-8.5 GHG emissions scenarios for BCC-CSM2-MR and SSP3-7.0 with GFDL-ESM4 (SSP5-8.5 remained unavailable) to estimate future ecosystem conditions at mid-century (2041–2060) and end-of-century periods (2081–2100; https://www.worldclim.org/data/cmip6/cmip6_clim2.5m.html). We modeled historical and future ecosystem distributions with a machine learning ensemble approach. Ensemble models often improve model fit and lower prediction error in comparison with individual model types^16^. We developed ML ensembles using a set of ‘base learners’ requiring only modest parameter tuning to avoid excessive computation time and overfitting^51,52^. We used four ML approaches known to produce robust results, namely gradient boosted (GBM), extreme gradient boosted (XGBT), extreme gradient boosted linear (XGBL) and random forest (RF) regression tree models. The first three models use “boosting” to assess and focus on model error at each of several model training iterations. The extreme boosting models include additional regularization steps and model tuning parameters to reduce the influence of weak predictors for obtaining parsimonious and generalizable model solutions^53^. Random forest applies “bagging” and multiple model iterations running in parallel to test predictors, and ultimately uses an aggregated voting process to select predictors showing the lowest model error^54^. A consolidated meta-model combined each technique or component model and used model weights based on the Root Mean Squared Error performance measure, for making predictions with a generalized gradient boosted model (‘gbm’). We used the caret v. 6.0.92 and caretEnsemble v. 2.0.1 packages in R statistical software for the model training, testing and prediction^55,56^.

We trained the distribution models for each ecosystem type by creating a polygon envelope encompassing all the spatially referenced occurrence records for that ecosystem. A random starting allocation of 2500 pseudo-absence points were located within the polygon envelope, centered on each occurrence record (100 km radius), constrained to the 5-state area. Each polygon envelope encompassed a broad range of conditions so that pseudo-absence locations were principally outside the range of suitable climate conditions but were in the same proximate portion of the study region as GAP occurrences.

The amount of presence and absence records were similar in sample size but left unbalanced, to improve model predictability^57^. This procedure allows absence locations to potentially occur within the minimum distance of one grid cell (approximately 4 km). The presence and absence data were combined and intersected with the historical bioclimate and terrain layers. Only a single presence or absence location per grid cell was allowed to eliminate sample redundancy (presence superseded absence). For each ecosystem, we used a random selection containing 80% of data for training the ensemble models and the remaining 20% for model validation.

Feature selection for optimizing predictor variables can improve distribution model performance^58^. Therefore, prior to model development we applied recursive feature elimination (RFE), a backward feature extraction method used to reduce and optimize predictor variables for each ecosystem type^59^. We implemented random forest tree functions (‘rfFuncs’) in the R caret package to rank predictors important to ecosystem distribution models. We considered the point at which the minimum root mean squared error (RMSE) was reached to select an optimized set of model predictors, which were used to fit each ML model in the ensemble.

We developed ‘historical’ ecosystem suitability models (1970–2000) using the 2.5-min scale historical bioclimate layers, producing the foundational ecosystem and bioclimate relationships. Model training included tenfold cross validation with bootstrap training data for parameterization. Validation data, omitted from model training, was used to assess RMSE, the receiver operating characteristic (ROC) for estimating area under the curve (AUC), and the Sørensen Similarity Index (SOR), calculated as TP/(FN + 2TP + FP) where TP indicates true positives, FN represents false negatives, and FP are false positives. Model predictions with AUC values ≥ 0.75 and Sørensen Similarity Index ≥ 0.5 indicate highest performance. The performance thresholds are ours, informed by our results and the thresholds frequently used by the scientific community, as generally accepted thresholds remain undefined. We developed future projections with the bioclimate predictors (predicted values and their spatial location) based on the different GCMs employed. Lastly, variable importance from permutational RMSE was used to evaluate key predictor variables underlying the habitat suitability predictions in the ensemble models using the DALEX package v. 2.4.2 for R statistical software^60^. The magnitude of increased RMSE with a variable removed from the models was used as an indicator of its importance to ecosystem suitability. This approach advances our prior methods for predicting ecosystem and climate relationships over geographical space and time^4^.

We examined ecosystem shifts by using suitability predictions, on a per ecosystem basis, by calculating the proportional change in total ecosystem area. For each ecosystem, we calculated the amount of suitable area per pixel by multiplying the suitability value occurring within the pixel by pixel area, and summing those values. We calculated percent change in total area of ecosystem suitability (future total−historical total)/historical total). We also calculated the proportional change in elevation and UTM northing between historical and future predictions of ecosystem suitability. For these calculations, we filtered pixels with suitability values ≥ 0.33 (except for Inter-Mountain Basins Juniper Savanna which used suitability values ≥ 0.16), extracted the corresponding pixel value for elevation or northing, obtained the mean values and quantified the proportional changes.

We quantified suitability for all ecosystems within specific, individual land management properties (*n* = 52,565). We utilized spatial polygons identifying these locations from the USGS Protected Areas Database (PAD-US) v. 3.0 (https://www.usgs.gov/programs/gap-analysis-project/science/pad-us-data-download). We also made predictions of ecosystem suitability for the Colorado Plateau Pinyon Juniper Woodland within locations subdivided by state boundaries, ownership (e.g., State, Federal, Tribal, and private) and properties managed by the National Park Service (NPS), United States Forest Service (USFS), Bureau of Land Management (BLM) and United States Fish and Wildlife Service (USFWS). For this exercise, we examined multiple GCMs (*n* = 9) with a wide range of climate sensitivities and SSP2-4.5, SSP3-7.0 and SSP5.85, to examine climate model uncertainty and geographical changes in future ecosystem suitability, given different model assumptions and rates of climate warming (Supplementary Table 3).

### Supplementary Information

Supplementary Figure 1.

Supplementary Figure 2.

Supplementary Figure 3.

Supplementary Figure 4.

Supplementary Table 1.

Supplementary Table 2.

Supplementary Table 3.

## Data Availability

All predictions of ecosystem suitability within the southwest United States based on climate change predictions using the Coupled Model Intercomparison Projects generation 6 (CMIP6), BCC-CSM2-MR and GFDL-ESM4 climate models, that incorporate the Shared Socioeconomic Pathways (SSP) 3–7.0 and SSP5-8.5 emissions scenarios are located here: https://ecos.fws.gov/ServCat/Reference/Profile/150238. Please cite the following DOI # for these data: https://doi.org/10.7944/P99PTGP4.
