# Supplementary material for: Climate change and ecosystem shifts in the southwestern United States
Source: Sci Rep. 2023 Nov 15;13:19964. doi: 10.1038/s41598-023-46371-x (PMC10651835; doi:10.1038/s41598-023-46371-x)
Supplement: Supplementary file 2 — Supplementary Figure 2. [file 41598_2023_46371_MOESM2_ESM.docx]

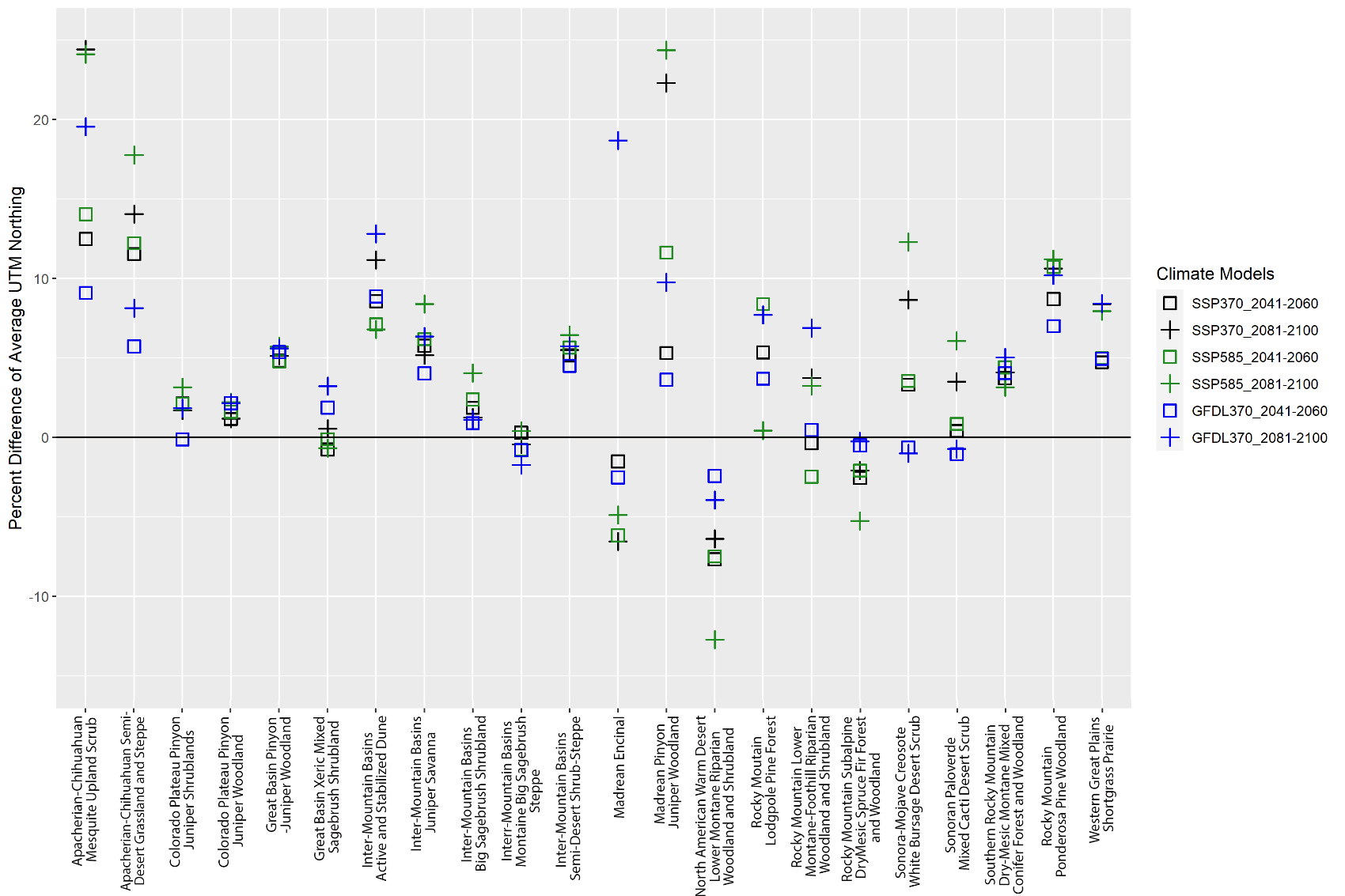


Supplementary Figure 2: Percent difference in UTM northings for 22 ecosystems within the southwestern USA. Percent difference calculations are based on differences in UTM northing values between the historic baseline and 6 future predictions occurring at mid-century (2041-2060; represented by squares) and end of century (2081-2100; represented with a "+"). These calculations consider pixels with suitability values > 0.33 (except for the Inter-Mountain Basins Juniper Savanna ecosystem which included values > 0.16). Data represent the following GCMs and emission scenarios: BCC-CSM2-MR SSP3-7.0 (SSP370, black), BCC-CSM2-MR SSP5-8.5 (SSP585, green), GFDL-ESM4 SSP3-7.0 (GFDL370, blue).
