# Supplementary material for: Climate change and ecosystem shifts in the southwestern United States
Source: Sci Rep. 2023 Nov 15;13:19964. doi: 10.1038/s41598-023-46371-x (PMC10651835; doi:10.1038/s41598-023-46371-x)
Supplement: Supplementary file 3 — Supplementary Figure 3. [file 41598_2023_46371_MOESM3_ESM.docx]

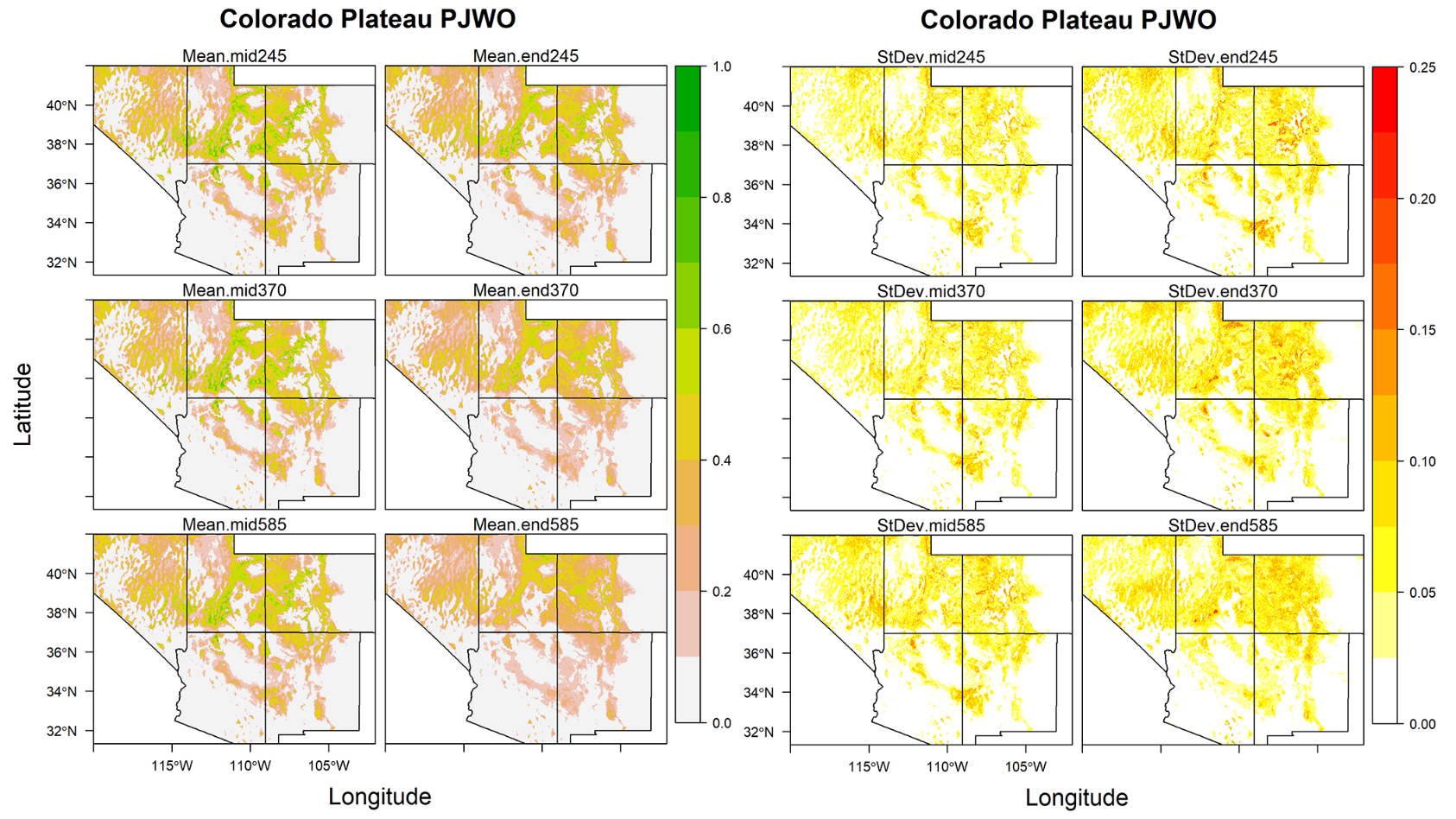


Supplementary Figure 3: Plots depicting the mean (right) and standard deviation (left) of ecosystem suitability on a per pixel basis (16.7 km^2^) for the Colorado Plateau Pinyon Juniper Woodland ecosystem within the southwestern United States (Nevada, Utah, Colorado, New Mexico, Arizona). Model projections rely on a suite of 9 climate models using the SSP2-45, SSP3-7.0 and SSP5-8.5 emission scenarios at mid-century (2041-2060) and end-of-century (2081-2100; Supplementary Table 3). Predictions of standard deviation indicate the amount of uncertainty in suitability projections across this region.
