# Supplementary material for: Climate change and ecosystem shifts in the southwestern United States
Source: Sci Rep. 2023 Nov 15;13:19964. doi: 10.1038/s41598-023-46371-x (PMC10651835; doi:10.1038/s41598-023-46371-x)
Supplement: Supplementary file 4 — Supplementary Figure 4. [file 41598_2023_46371_MOESM4_ESM.docx]

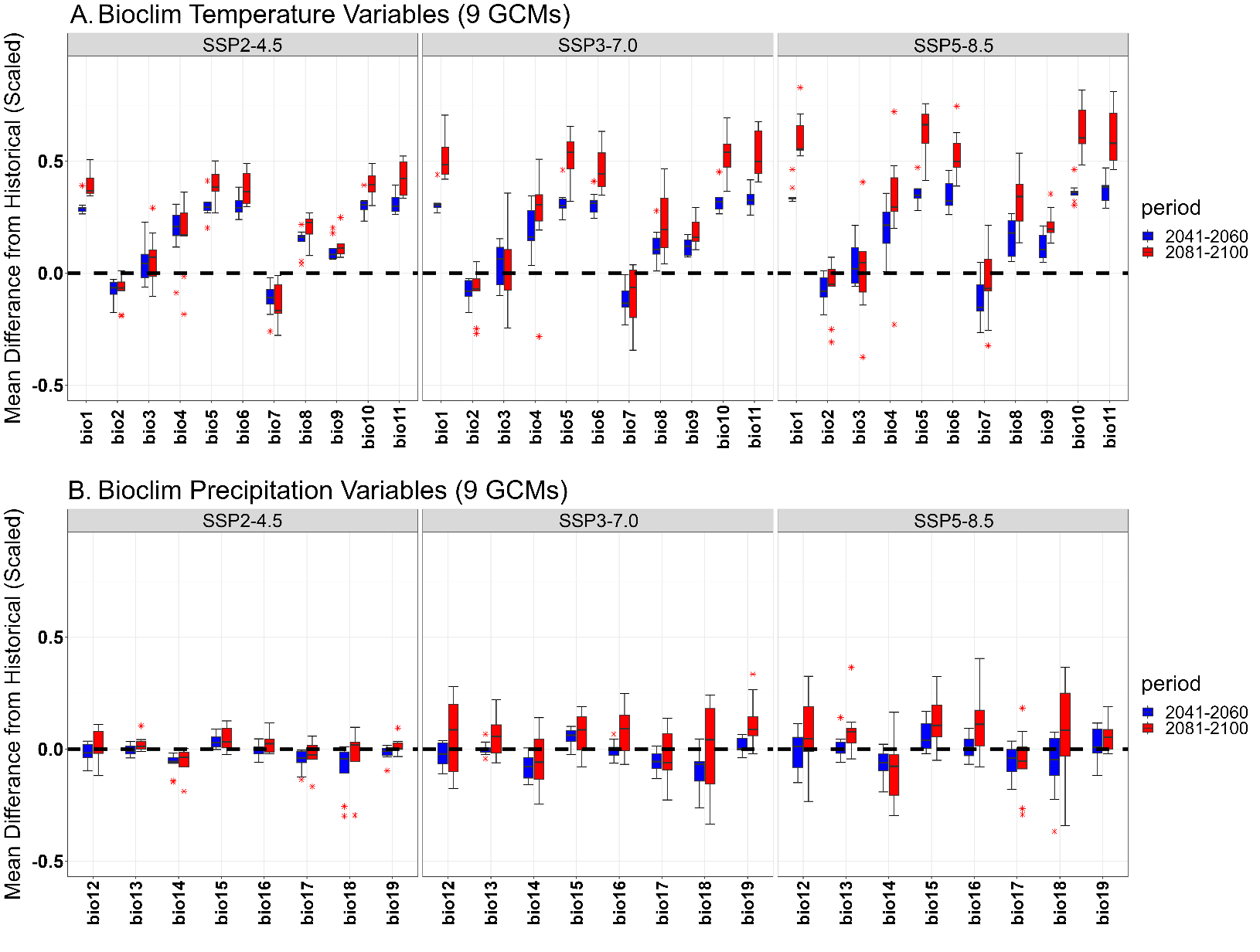
Supplementary Figure 4: Box and whisker plots for 19 bioclimate variables depicting the differences in mean values between historic (1970 – 2000) and two future periods (blue representing mid-century (2041 - 2060) and red indicating end-of-century (2081 - 2100)) for 9 global climate change models (GCMs, Supplementary Table 6) and three emission scenarios, covering the Colorado Plateau Pinon Juniper Woodland ecosystem. Bioclimate variables 1 – 11 (plot A) are associated with temperature while bioclimate variables 12 – 19 (plot B) are associated with precipitation (Table 2). Note the increases in most temperature values (positive mean differences) for each period, irrespective of emission scenario. Values for mean diurnal range (bio2), isothermality (bio3) and temperature annual range (bio7) indicate decreasing value ranges. Plots of precipitation variables reveal greater uncertainty among GCMs (mean difference values spanning 0), with similar patterns between them. For each bioclimate variable, values were calculated and scaled per the following: Future value – historic value, when the result is >= 0, the result is scaled using the difference value/maximum positive difference value, or if the result is < 0 then the result is scaled by taking the difference value/minimum negative difference value to retain the direction and magnitude of the difference.
