# Supplementary material for: Climate change and ecosystem shifts in the southwestern United States
Source: Sci Rep. 2023 Nov 15;13:19964. doi: 10.1038/s41598-023-46371-x (PMC10651835; doi:10.1038/s41598-023-46371-x)
Supplement: Supplementary file 7 — Supplementary Table 3. [file 41598_2023_46371_MOESM7_ESM.docx]

Supplementary Table 3: Climate models used in ensemble modeling for predicting and projecting ecosystem suitability for the Colorado Plateau Pinyon Juniper Woodland ecosystem from historical (1970-2000) geographical locations within the southwestern United States (Arizona, Colorado, Nevada, New Mexico, Utah).

| **Climate model (CMIP6)** | **Citation** | **Climate Sensitivity** °C **^1^** |
| --- | --- | --- |
| ACCESS-ESM1-5 | Ziehn et al. 2020 | 3.9 |
| BCC-CSM2-MR | Wu et al. 2019 | 3.0 |
| CNRM-CM6-1 | Voldoire et al. 2019 | 4.9 |
| CNRM-ESM2-1 | Séférian et al. 2019 | 4.8 |
| CanESM5 | Swart et al. 2019 | 5.6 |
| IPSL-CM6A-LR | Boucher et al. 2020 | 4.6 |
| MIROC-ES2L | Hajima et al. 2019 | 2.7 |
| MIROC6 | Tatebe et al. 2019 | 2.6 |
| MRI-ESM2-0 | Naoe and Yoshida 2019 | 3.1 |

^1^Equilibrium climate sensitivity (ECS) is the expected long-term warming after a doubling of atmospheric CO2 concentrations as a result of physical and biogeochemical simulations within climate models.

References

Boucher O., J. Servonnat, A.L. Albright, O. Aumont, Y. Balkanski, V. Bastrikov et al. 2020. Presentation and evaluation of the IPSL‐CM6A‐LR climate model. Journal of Advances in Modeling Earth Systems, 12, e2019MS002010. <https://doi.org/10.1029/2019MS002010>

Hajima, T., M. Watanabe, A. Yamamoto, H. Tatebe, M.A. Noguchi, M. Abe et al. 2020. Development of the MICRO-ES2L Earth system model and the evaluation of biogeochemical processes and feedbacks. Geoscientific Model Development 13: 2197-2244

Naoe, H. and K. Yoshida. 2018. Influence of quasi-biennial oscillation on the boreal winter extratropical stratosphere in QBOi experiments. Quarterly Journal of the Royal Meteorological Society 145: 2755-2771

Séférian, R. P. Nabat. M. Michou, D. Saint-Martin, A. Voldoire, J. Colin, B. Decharme, C. Delire, S. Berthet, M. Chevallier, S. Sénési, L. Franchisteguy, J. Vial, M. Mallet, E. Joetzjer, O. Geoffroy, J. Guérémy, M. Moine, R. Msadek, A., Ribes, M., Rocher, R. Roehig, D. Salas-y-Mélia, A. Sanchez, L. Terray. S. Valcke, R. Waldman, O. Aumont, L. Bopp, J. Deshayes, C. Éthé, and G. Madec. 2019. Evaluation of CNRM Earth‐System model, CNRM‐ESM2‐1: role of Earth system processes in present‐day and future climate. Journal of Advances in Modeling Earth Systems, 11: 4182-4227

Swart, N.C., J.N.S. Cole, V.V. Kharin, M. Lazare, J.F. Scinocca, N.P. Gillet, J. Anstey, V. Arora, J.R. Christian, S. Hanna, Y. Jiao, W.G. Lee, F. Majass, O.A. Saenko, C. Seiler, C. Seinen, A. Shao, M. Sigmond, L. Solheim, K. von Salzen, D. Yang, and B. Winter. 2019. The Canadian earth system model version 5 (CanESM5.0.3). Geoscientific Model Development. 12: 4823-4873

Tatebe, H., T. Ogura, T. Nitta, Y. Komuro, K. Ogochi, T. Takemura et al. 2019. Description and basic evaluation of simulated mean state, internal variability, and climate sensitivity in MICRO6. Geoscientific Model Development 12: 2727-2765

Voldoire, A., D. Saint-Martin, S. Sénési, B. Decharme, A. Alias, M. Chavellier, J. Colin, J.-F. Guérémy, M. Michou, M.-P. Moine, P. Nabat, R. Roehrig, D. Salas y Mélia, R. Sérférian, S. Valcke, I. Beau, S. Belamari, S. Berthet, C. Cassou, J. Cattiaux, J. Deshayes, H. Douville, C. Ethé, L. Franchistéguy, O. Geoffroy, C. Lévy, G. Madec, Y. Meurdesoif, R. Msadek, A. Ribes, E. Sanchez-Gomez, L. Terray, and R. Waldmand. 2019. Evaluation of CMIP6 DECK experiments with CNRM‐CM6‐1. Journal of Advances in Modeling Earth Systems, 11: 2177–2213

Wu, T., Y. Lu, Y. Fang, X. Xin, L. Li, W. Li, W. Jie, J. Zhang, Y. Liu, L. Zhang, F. Zhang, Y. Zhang, F. Wu, J. Li, M. Chu, Z. Wang, X. Shi, X. Liu, M. Wei, A. Huang, Y. Zhang, and X. Liu. 2019. The Beijing climate center system model (BCC-CSM): the main progress from CMIP5 to CMIP6. Geoscientific Model Development. 12: 1573-1600.

Ziehn, T., M.A. Chamberlain, R.M. Law, A. Lenton, R.W. Bordman, M. Dix, L. Stevens, Y. Wang, and J. Srbinovsky. 2020. The Australian earth system model: ACCESS-ESM1.5. Journal of Southern Hemisphere Earth Systems Science. 70: 193-214.
